# Photoproximity Labeling from Single Catalyst Sites Allows Calibration and Increased Resolution for Carbene Labeling of Protein Partners In Vitro and on Cells

**DOI:** 10.1021/acscentsci.3c01473

**Published:** 2023-12-26

**Authors:** Thomas G. Bartholow, Paul W.W. Burroughs, Susanna K. Elledge, James R. Byrnes, Lisa L. Kirkemo, Virginia Garda, Kevin K. Leung, James A. Wells

**Affiliations:** † Department of Pharmaceutical Chemistry, University of California San Francisco, San Francisco, California 94158, United States; ⊥ Department of Cellular & Molecular Pharmacology, University of California San Francisco, San Francisco, California 94158, United States

## Abstract

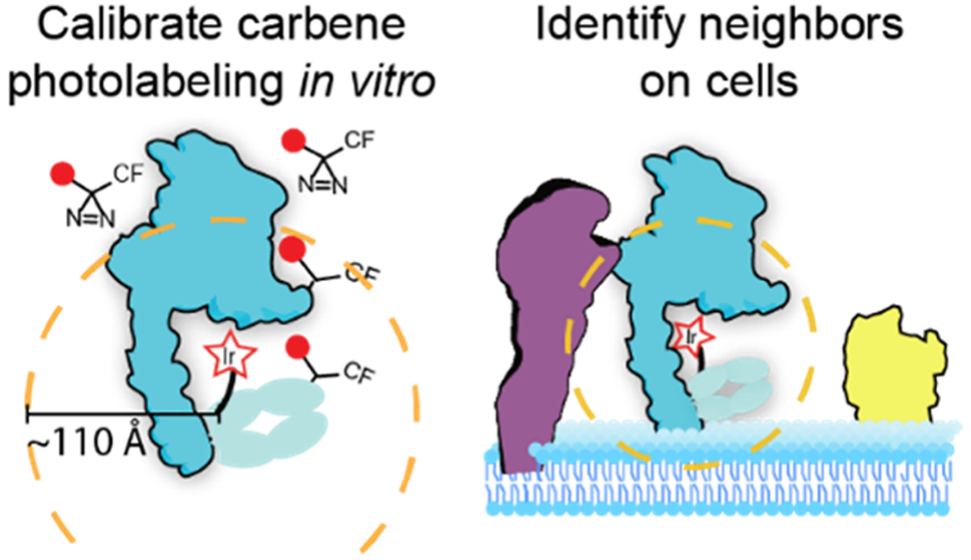

The cell surface proteome (surfaceome) plays a pivotal role in virtually all extracellular biology, and yet we are only beginning to understand the protein complexes formed in this crowded environment. Recently, a high-resolution approach (μMap) was described that utilizes multiple iridium-photocatalysts attached to a secondary antibody, directed to a primary antibody of a protein of interest, to identify proximal neighbors by light-activated conversion of a biotin–diazirine to a highly reactive carbene followed by LC/MS (Geri, J. B.; Oakley, J. V.; Reyes-Robles, T.; Wang, T.; McCarver, S. J.; White, C. H.; Rodriguez-Rivera, F. P.; Parker, D. L.; Hett, E. C.; Fadeyi, O. O.; Oslund, R. C.; MacMillan, D. W. C. *Science***2020**, *367*, 1091–109710.1126/science.aay4106PMC733666632139536). Here we calibrated the spatial resolution for carbene labeling using site-specific conjugation of a single photocatalyst to a primary antibody drug, trastuzumab (Traz), in complex with its structurally well-characterized oncogene target, HER2. We observed relatively uniform carbene labeling across all amino acids, and a maximum distance of ∼110 Å from the fixed photocatalyst. When targeting HER2 overexpression cells, we identified 20 highly enriched HER2 neighbors, compared to a nonspecific membrane tethered catalyst. These studies identify new HER2 interactors and calibrate the radius of carbene photoprobe labeling for the surfaceome.

## Abstract 2

Our work optimizes and calibrates the maximal distance for photocatalytic carbene labeling to identify the most proximal protein neighbors for cell surface proteins, specifically the oncogene HER2.

## Introduction

The human cell surface proteome, or surfaceome, is encoded by one-fourth of the genome and represents the cellular interface for communication with other cells including signaling, cell nutrient transport, and cell–cell contact.^1−3^ The surfaceome is also the target of virtually all biologics and half of small molecule drug targets.^4^ Thus, in recent years there has been a surge of mass spectrometry-based proteomics approaches to probe how the surfaceome is remodeled from health to disease, in terms of protein expression,^2,5−7^ post-translational modifications (PTMs) such as glycosylation,^8−11^ and proteolysis.^12^ However, these approaches have not interrogated changes in the landscape and dynamics of protein complexes that initiate cell signaling.

In the past decade, the field of protein interactomics has made huge advances to identify stable protein complexes within the 3D environment of cells, principally with affinity tagged pull-down mass spectrometry.^13−18^ The success of this approach requires high-affinity binding between partners for interactions to survive the pull-down workup. However, membrane protein interactions are not always captured in pull-down methods, due to intrinsically lower affinity interactions because in 2D membrane proteins they have lower entropic freedom.^19−21^

To address this problem, the field of proximity labeling proteomics (PLP) has emerged as a way to take covalent snap-shots of proteins *in situ* by localized generation of reactive species containing a covalent biotin affinity handle for purification.^22,23^ Peroxidase-based PLP, such as ascorbate peroxidase (APEX),^24^ have pioneered our ability to label the local proteomes of large structures like synapses^25^ and cilia^26^ within minute time-scales. However, peroxidases generated hydroxy radicals are long-lived (*t*_1/2_ ∼ 0.1 ms) and estimated to diffuse large distances, up to 3000 Å.^27^ This labeling distance is more than 10 times the dimensions of typical binary protein complexes and is prone to capture bystander proteins in the crowded surfaceome. Chemical cross-linking is a powerful high-resolution alternative for structural characterization of strong complexes or dynamic protein states,^28^ but necessitates very close proximity of particular cross-linked residues (typically, two lysines within about 15–30 Å). Such cross-linking reactions are challenging to analyze from complex cellular samples and so have been mostly limited to purified complexes.^29^ Thus, there remains a gap for probing transient protein–protein interactions in complex cellular milieus, especially membrane proteomes.

In a significant step forward, the Oslund, Fadeyi, and MacMillan groups recently described a higher resolution PLP approach, called μMap.^30^ μMap locally generates short half-lived carbenes (*t*_1/2_ ∼ 2–4 ns) from biotinylated diazirine compounds, through illumination of a protein bound iridium catalyst and Dexter Energy Transfer (DET). Carbenes are known to insert into C–H bonds, among others, and indiscriminately label a broad spectrum of amino acid types.^31,32^ μMap was shown to label cell surface complexes with higher resolution estimated to have a range of ∼500–600 Å.^30^ However, the μMap method placed six to eight Ir-based catalysts by a random *N*-hydroxysuccinamide (NHS) labeling of lysines onto a secondary antibody that binds a primary antibody directed to the membrane protein of interest.

Here, we calibrated the carbene labeling distances from photocatalyst sites by site-specific conjugation onto the primary antibody to a target protein and measured intramolecular and intermolecular carbene labeling distances *in vitro*. For the test system, we used Fabs derived from the therapeutic antibody trastuzumab (Traz), whose structure is known in complex with its drug target, HER2.^33^ Through *in vitro* mass spectrometry studies, we confirmed broad labeling of amino acids at single amino acid resolution and demonstrated that labeling reaches a maximum distance of ∼110 Å from the Ir-catalyst site. Furthermore, to improve the confidence in partners identified on cells, we determined enriched proteins relative to a nonspecific membrane bound Ir-catalyst control as opposed to the catalyst on the secondary antibody in solution. More than a dozen new candidate interacting partners for HER2 were discovered with strong functional links to breast cancer. These studies serve to calibrate the distance dependence for carbene labeling and provide greater spatial resolution and confidence for identifying membrane neighbors.

## Results

### Experimental Strategy

We chose the Traz–HER2 system for in-depth analysis because it is structurally well characterized and is of therapeutic interest. Moreover, Traz binds but does not impair signaling^35^ so would allow us to identify new partners near the on-state form of HER2. Moreover, we have previously shown that the Fab for Traz is highly amenable to single site-specific labeling using engineered methionines and their specific reaction with oxaziridines for stable and selective bioconjugation.^34^

**Figure 1:**
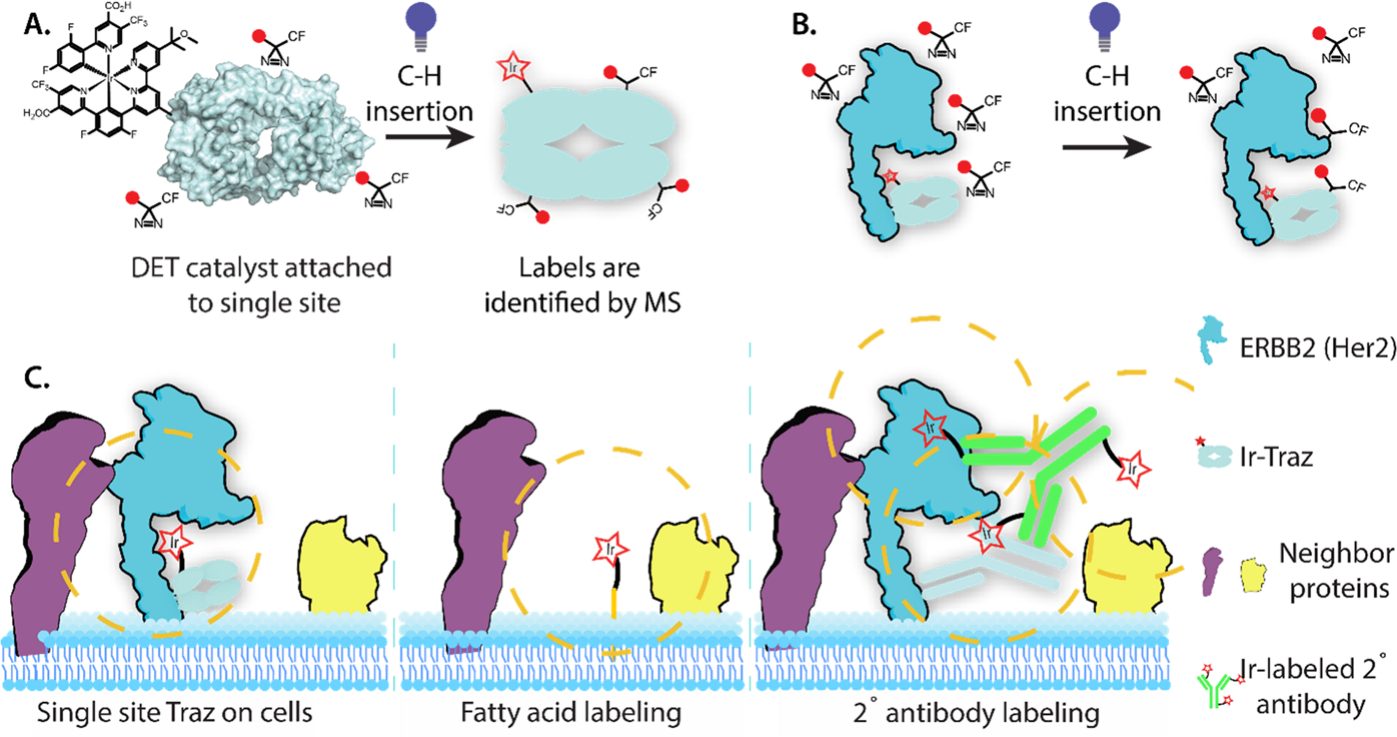
Experimental strategy to probe intramolecular, intermolecular, and on-cell biotin–carbene labeling. A. Intramolecular biotin–carbene labeling for Ir-catalysts attached at single sites on the Traz-Fab. Labeling is catalyzed upon blue light illumination, which triggers localized biotin–carbene generation from free diazirine. Quantitative mass spectrometry was used to determine the precise sites labeled by biotin–carbenes. These sites, mapped onto the known structure (PDB: 1N8Z), were used to assess distance dependence. B. Intermolecular labeling from the Fab-catalyst to HER2 in the complex was performed using the same procedures as in A, to assess distance dependence from different Fab-catalyst probes. C. On-cell biotin–carbene labeling of cells expressing HER2 bound with specific Fab-catalysts (left) or secondary IgG catalyst (right) compared to the FA-catalyst nonspecific control (control). HER2 is in blue and hypothetical partner in purple and bystander in yellow.

We divided our studies into three parts (Figure 1). First, we interrogated intramolecular self-labeling *in vitro* by installing single Ir-photocatalysts at eight different sites in both the VH and CH-1 domains of heavy and light chain in the Fab. Here we sought to understand how biotin–carbene labeling patterns varied within the Fab as the attachment site for the Ir photocatalyst varied. Using high resolution mass spectrometry, we evaluated (i) the precise sites of intramolecular carbene labeling within the Fab, (ii) the distribution of residue type labeled, and (iii) the distance dependence from the catalyst site to labeled amino acid by inspection of the crystal structure (PDB: 1N8Z) (Figure 1A).

Next, we determined intermolecular target labeling characteristics from the eight Fab Ir-photocatalyst bound to HER2 in vitro by analyzing carbene labeling patterns using mass spectrometry (Figure 1B). Finally, we determined the on-cell HER2 interactome using the different Fab-catalysts bound to HER2 on SKBR3 cells.^36^ SKBR3 cells are a well-known breast cancer cell line that overexpresses HER2 leading to constitutive oncogenic activation (Figure 1C). These on-cell analyses were benchmarked against a reference NHS randomly labeled secondary antibody strategy used by μMap. As a nonspecific cellular control, we also created an Ir-catalyst attached by click-chemistry to an azido-fatty acid (FA) that readily inserts into the cell membrane. This FA-catalyst control nonspecifically sampled the membrane proteomic background, to which both primary and secondary antibody catalysts could be quantitatively compared for higher confidence interactome identification. We also show the FA-catalyst control can also serve as a general approach for membrane PLP.

### Analysis of Intramolecular Labeling of the Traz-Fab from the Ir-Photocatalyst Positioned at Eight Sites

To create the Fab-catalysts, we expressed eight different single surface Met variants among 60 sites previously shown to be robustly expressed and quantitatively labeled with an oxaziridine for click chemistry.^34^ Sites chosen were distributed throughout both light and heavy chains, in both the VH domain proximal to the Fab’s CDRs, as well as in the CH1 domain distal from the CDRs (Supporting Information Figure 1A). These eight Met Fab variants were expressed into the periplasm of *E. coli* and purified to >95%; each was labeled to >50% at the engineered single Met site using an oxaziridine–azide reagent via a sulfamide linkage. Stoichiometry was confirmed by mass spectrometry (Supporting Information Figure 2A).^34^ The Traz-Fab used contains three buried Mets, which have been shown not to react with the oxaziridine-azide reagent.^34^ We next coupled the Ir-catalyst using copper-free click chemistry;^37^ the final conjugated structure has a maximal extended linker length of ∼50 Å (Supporting Information Figure 1B).

We employed biotin–diazirine labeling using conditions similar to those for μMap,^30^ where the Fab-catalyst concentration was set at 9 μM, and the biotin–diazirine concentration at 100 μM (11-fold excess). Conjugates were illuminated with blue light for 5 min at 25 °C in phosphate-buffered saline (PBS). The extent of labeling is controlled by the concentration of the biotin–diazirine, ratio of biotin–diazirine to protein, and time of illumination.

**Figure 2:**
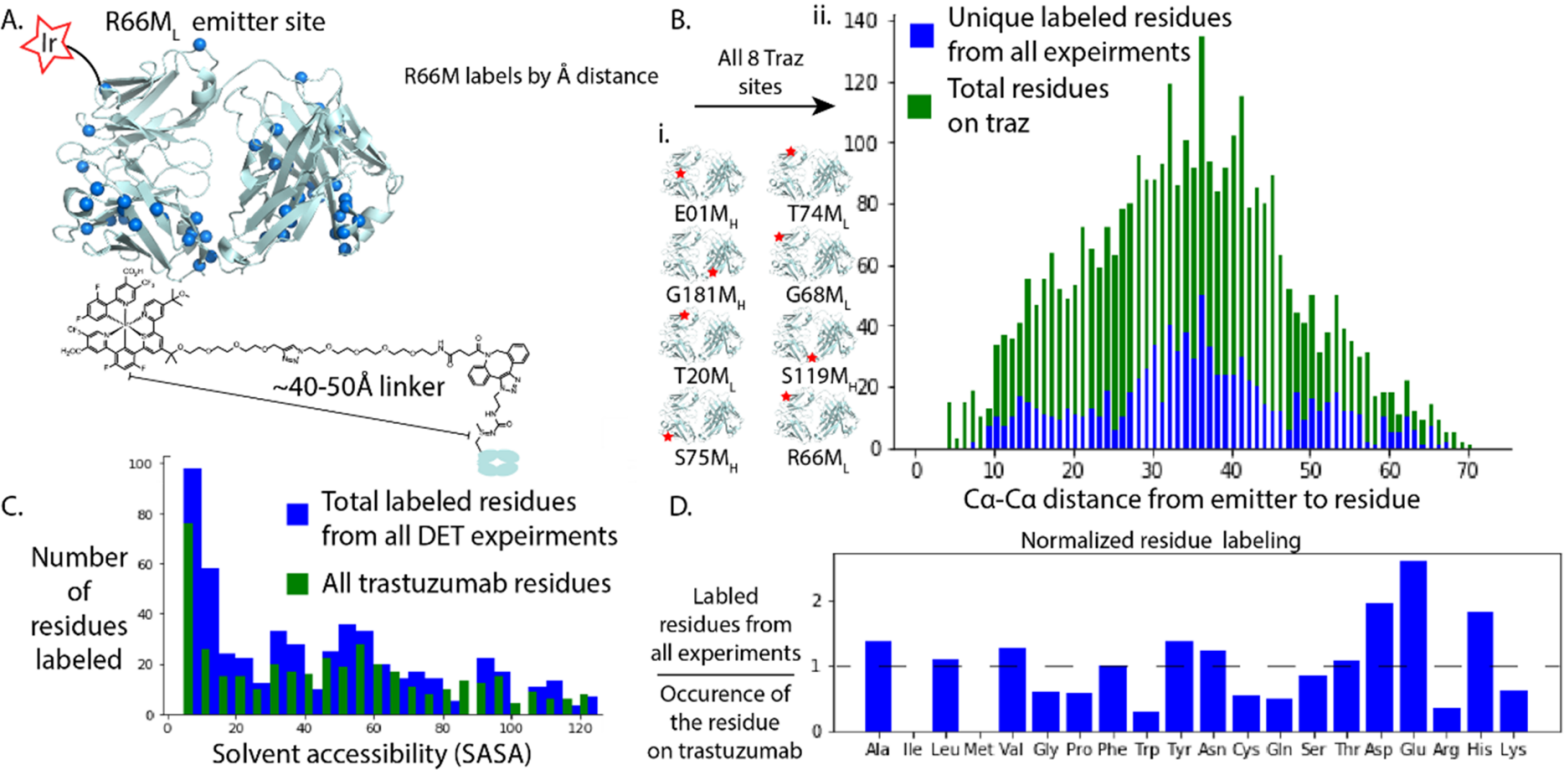
Dependence of distance, solvent accessibility, and residue type for intramolecular biotin–carbene labeling for eight Fab-catalyst probes. A. Ribbon diagram for a single Fab-catalyst (R66M_L_) where the Ir-catalyst was attached (red star). Locations of biotin–carbene labels are shown as blue dots. B.i. Ribbon diagrams for all eight Fab-catalysts with red stars showing the locations of the Ir-catalyst on the Fab, with specific mutation site indicated below. ii. Blue bars show a histogram for the Cα-to-Cα distance from the Ir-catalyst to sites of carbene labeling, compiled for all eight Fab-catalysts. Note the Gaussian shape centered at 30–40 Å, and that labeling extends to the 65 Å limit of the Fab’s longest dimension. Green bars show a histogram for Cα-to-Cα distance from the Ir-catalyst to all amino acids in the Fab. Individual plots for each emitter are available in Supporting Information Figure 2D. C. SASA values (calculated by the Shrake–Rupley algorithm) for the labeled residues identified in all experiments (blue bars) and total residues of Traz (green bars). The ratio of green to blue is relatively consistent, suggesting that labeling can occur if residue is partially or fully exposed. Due to the small size of Traz, every residue has some solvent accessibility. D. Normalized frequency of carbene labeling for the 20 amino acids, with a dotted line noting a 1:1 ratio. Normalization was accomplished by taking the unique carbene labels onto each amino acid, divided by the frequency of that specific amino acid present in Traz. Correcting for greater occurrence of certain amino acids on Traz resulting in values over 1.0. The data suggest labeling is somewhat uniform.

We then analyzed the sites of intramolecular biotin–carbene labeling in three biological replicates, each with two technical replicates, for all eight Fab-catalysts at single amino acid resolution via mass spectrometry (Figure 2). Samples were digested with trypsin, with resulting peptides processed with a Preomics mass spectrometry prep kit.^38^ Tryptic peptides were injected onto a Bruker TimsTOF Pro. Data were processed using the PEAKS informatics program.^39^ The location of the individual residues was readily identified by the presence of an additional monoisotopic mass of 616.25, corresponding to insertion of the biotin–carbene. Given the low sample complexity of tryptic peptides from these purified proteins, it was not necessary to enrich the biotinylated peptides. Total coverage of peptides from the light chain was 98.6%, and for the heavy chain was 73.9%, when processed using a <1% FDR. While the coverage is very high, the tryptic peptides, S140–K153, and terminal S196-G239 of the Traz heavy chain were not detected.

Each Fab-catalyst was covalently labeled with the biotin–containing probe at an average of 74 individual sites (± 10), out of about 434 amino acid positions possible in the combined light and heavy chain. The precise sites of self-labeling were mapped onto the structure of the Fab-catalysts (Figure 2A; Supporting Information Figure 2C,D). There was a large degree of overlap for the labeling patterns for the eight Fab-catalysts, although each was unique, as seen by the Venn diagrams in Supporting Information Figure 2B.

From inspection of the crystal structure of Traz,^33^ we determined the Cα to Cα distance from the methionine containing the Ir-catalyst to each biotin–carbene labeled amino acid for all of the eight Fab-catalysts (blue bar graph shown in Figure 2B). Summing the number of labels at each distance (based on ∼500 total observations) produced a Gaussian distribution centered around 30–40 Å. We compared this to a plot of the distances between the conjugated site and all residues (labeled or not) shown by the green bar graph overlaid in Figure 2B. This plot reflects the opportunity to label and shows a remarkably similar Gaussian distribution centered around 30–40 Å. If we then take the ratio of these two, for each distance position, this produced a rather flat plot that extends to the very edge of the Fab at 65 Å. The decrease in labeling at the lower and higher angstrom distances matches the lower number of potential sites for labeling. We interpret this to mean that labeling potential is rather uniform up to the limit of the Fab, ∼65 Å.

We next analyzed the normalized frequency of labeling by amino acid type (Figure 2D). In aggregate, the labeling was broadly distributed across amino acid types, and the labeling ratio varied only about ±2-fold from the mean. There are ∼20 residues that are almost entirely buried in the Fab, for which we did not see labeling, including all Ile and Met, which explains their lack of representation. We next determined how solvent-accessible surface area (SASA) correlated with biotin–carbene labeling using standard Shrake–Rupley solvent accessibility calculations^40^ (Figure 2C). We found that carbene labeling could occur at all residues from partial to complete exposure (green bars), and this mirrored the average distribution of SASA for residues in the Fab (blue bars). Interestingly, we found partially buried residues had increased labeling frequency of carbene labeling possibly because they are more protected from competing hydrolysis. Taken together, these data demonstrate that there is little bias for the residue type that is labeled, so long as they are partially solvent accessible and that labeling is rather uniform to the edge of the Fab.

### Analysis of Intermolecular Labeling from Fab-Catalysts to HER2 in the Complex

To evaluate the biotin–carbene labeling patterns for the Fab-catalysts in complex with the purified ecto-domain of HER2, we mixed them 1:1 (9 μM each) and then conducted the biotin–diazirine photolabeling. Given the high affinity of the Traz Fab for HER2 (*K*_D_ ∼ 2 nM),^41^ we predicted that >95% of partners should be in a 1:1 steady-state complex. The mass spectrometry workup and analysis was the same as for the Fab-catalyst self-labeling experiment above.

**Figure 3:**
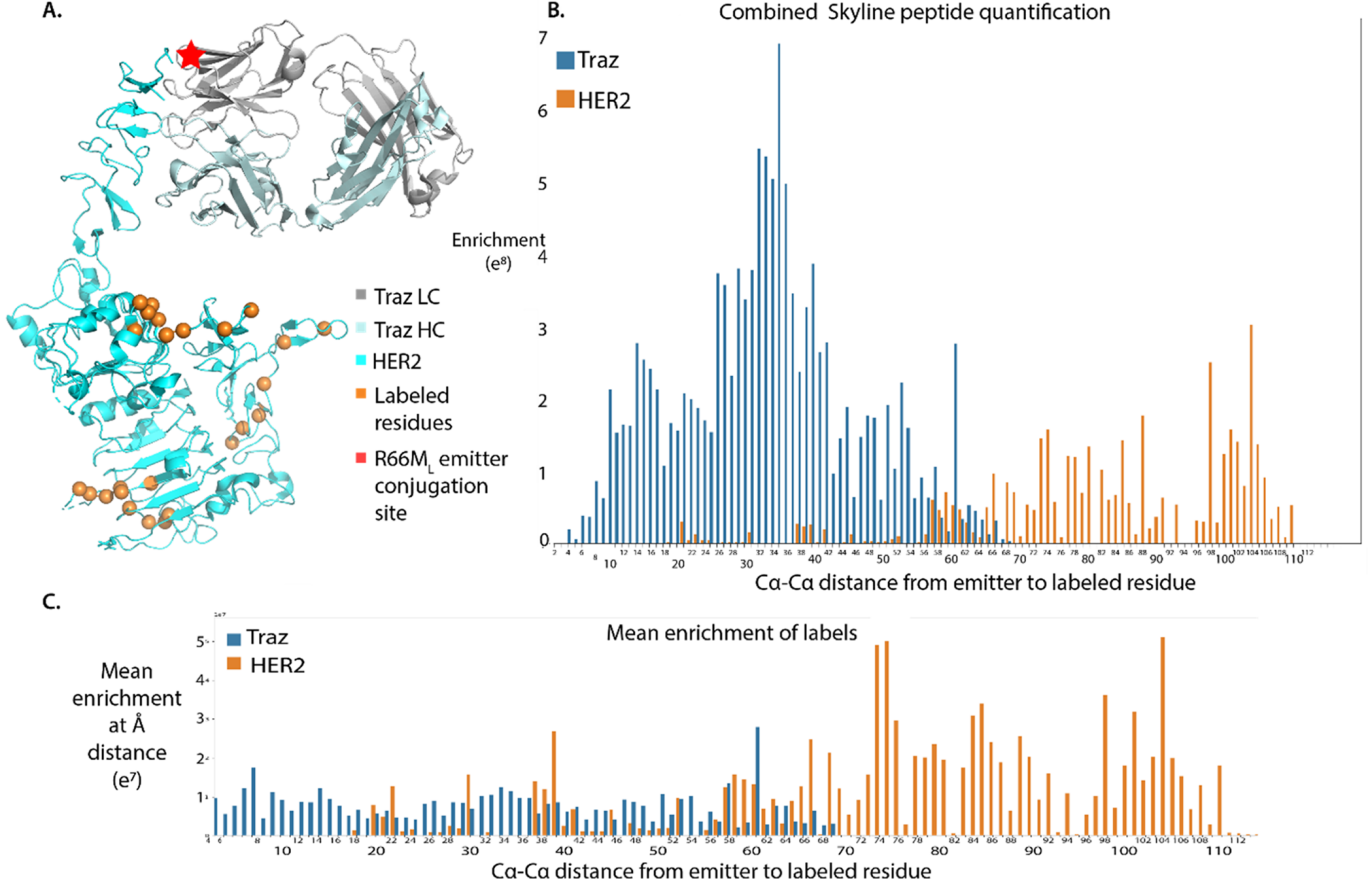
Intermolecular biotin–carbene labeling from Fab-catalyst in complex with HER2. A. Example of biotin–carbene labeling of HER2 using the R66M_L_ Fab catalyst. HER2 is rendered in cyan ribbons, and Cα positions labeled are shown as orange spheres. The example R66M_L_ Fab-catalyst is shown in gray and teal ribbons, with the site of the Ir-catalyst shown as a red star. B. The total Cα-to-Cα distance distribution plot for intramolecular and intermolecular labeling, compiled for all eight Fab–HER2 complexes, showing the labeling of the Fab-catalysts (blue bars) and HER2 (orange bars). Labeling is graphed by enrichment calculated by Skyline. C. Enrichment of labeled residues at each distance from the Ir-catalyst, taken as the average of enrichment at each angstrom distance.

We evaluated how the self-labeling patterns for the Fab-catalysts changed when bound. The Fab-catalysts showed lower extents of self-labeling, with an average of 55 sites (±11) when bound to HER2, compared to an average of 74 sites (±10) when free. Some of this reduction may be because the HER2 complex is consuming available carbenes, which could then be limiting. In addition, we see evidence that the CDRs can be partially protected from labeling when bound to HER2, as shown for the R66M_L_ Fab-catalyst in Figure 3A.

We next analyzed the intermolecular labeling patterns on HER2. Peptide coverage for HER2 averaged 52% with FDRs < 1%; the “neck” region of HER2 is highly hydrophobic and poorly cleaved by Trypsin/LysC, likely explaining why peptides from this region were not resolved by MS (red ribbons in Figure 3B). Each Fab-catalyst labeled between 11 to 28 sites on HER2, with an average of 16 sites (±12). The Fab-catalysts labeled many of the same sites on HER2 as shown by Venn diagrams in Supporting Information Figure 3A,B, but each had a unique labeling pattern; this is not surprising given their different locations.

Next, we determined the distance dependence of labeling for the complex, both intramolecular labeling of the Fab-catalyst alone, and intermolecular labeling of the Fab-catalyst complexed to HER2 (Figure 3C). For HER2, we see about 10 times lower intensity of labeling than for the self-labeling by the Fab-catalyst. The labeling on HER2 extends from as close as ∼20 Å from the closest Fab-catalyst to ∼110 Å to the most distal. The furthest labeling distance possible on HER2 is ∼120 Å.

We analyzed the extent of labeling versus distance from the Ir-catalyst for all eight complexes. For residues within ∼80 Å of their most distant catalyst site, the extent of labeling from different catalysts was very similar and generally flat ≥ (Supporting Information Figure 4). However, for those sites with catalysts further than 100 Å from the carbene labeled site, there was a sharp drop-off in extent of labeling for the more distal residues consistent with a maximum labeling near ∼110 Å.

### Labeling of HER2 and Its Neighborhood on SKBR3 Cells

We next tested the ability of each of the eight Fab-catalysts to label HER2 and neighbors on a well-characterized breast cancer-derived cell line, SKBR3, where HER2 is overexpressed (1.6 million receptors per cell), leading to constitutive self-activation.^36^ Each of the eight Fab-catalysts were added, at 5 μg/mL (120 nM), to 5 million SKBR3 cells for 30 min at 4 °C. Excess Fab was quickly washed away followed by labeling with 100 μM diazirine–biotin for 10 min of blue light exposure. We deliberately chose 4 °C because it is below the lipid fluid phase transition, thus effectively quenching any further trafficking or diffusion on the membrane. Following labeling, cells were washed and lysed to capture biotinylated proteins to be analyzed by mass spectrometry. Each Fab-catalyst sample was analyzed in biological triplicate and technical duplicate. Maxquant was used for processing on-cell experiments, with Perseus used for further analysis. Each Fab-catalyst alone captured an average of 356 proteins (±21) (Supporting Information Table 3). Together, the eight catalysts captured 742 proteins with ≥2 unique peptide per protein identified. About 90% of captured proteins were annotated to be plasma membrane proteins based upon combination of SwissProt GOCC Plasma membrane and Uniprot databases (Supporting Information Figure 5).

To rigorously compare proteins specifically labeled by the Fab-catalysts on the cell surface, we engineered a nonspecific membrane catalyst through conjugation of the Ir-catalyst to a membrane anchored fatty acid. Five million SKBR3 cells containing the FA-catalyst were analyzed in biological triplicate with two technical replicates as above. The FA-catalyst identified a total of 787 proteins with high confidence (false discovery rate (FDR) < 1%) (Supporting Information Table 1).

To further validate the FA-catalyst’s ability to act as a less biased surfaceome control, we compared its labeling pattern to the well-established method of cell surface capture (CSC). The CSC approach captures the glycoprotein population of the cell surface, representing ∼85% of membrane proteins.^2^ Using the same number of cells, we identified 543 proteins by CSC, with a standard deviation (SD) of 0.39 log2x units between biologic replicates, compared to 787 proteins found by the FA-catalyst with an SD of 0.32 SD. There was substantial overlap with proteins identified by the FA-catalyst and CSC in both quantity and proteins identified (Supporting Information Figure 8; Supporting Information Table 1). The small differences we find are likely due to the different cell surface labeling methods: the FA-catalyst labels anything within ∼110 Å, while CSC is restricted to glycoproteins. Taken together, we believe this data validates the FA-catalyst for use in cell surfaceome labeling. Importantly, it allows us to compare methods (Fab vs FA) that utilize the same Ir-based catalyst bound to the cell membrane proteins or lipid bilayer, respectively.

Next, we sought to compare our site-specific Fab-catalyst with the nonspecific NHS labeling of the original μMap method.^30^ The μMap method used a mouse Fc secondary antibody labeled with roughly 6–8 Ir-catalysts. We produced this and tested labeling from solution as described in the μMap method and identified only about 150 proteins with SD of 0.67, compared to 787 proteins for the FA-catalyst and an SD of 0.32 (Supporting Information Table 1). Virtually all of the 150 proteins identified by the secondary IgG-catalyst control were identified in the FA-catalyst control as shown by the Venn diagram (Supporting Information Figure 5C). These data support that the FA-catalyst more rigorously controls for nonspecific membrane labeling of Fab-catalysts on the membrane than a solution borne antibody catalyst control.

**Figure 4:**
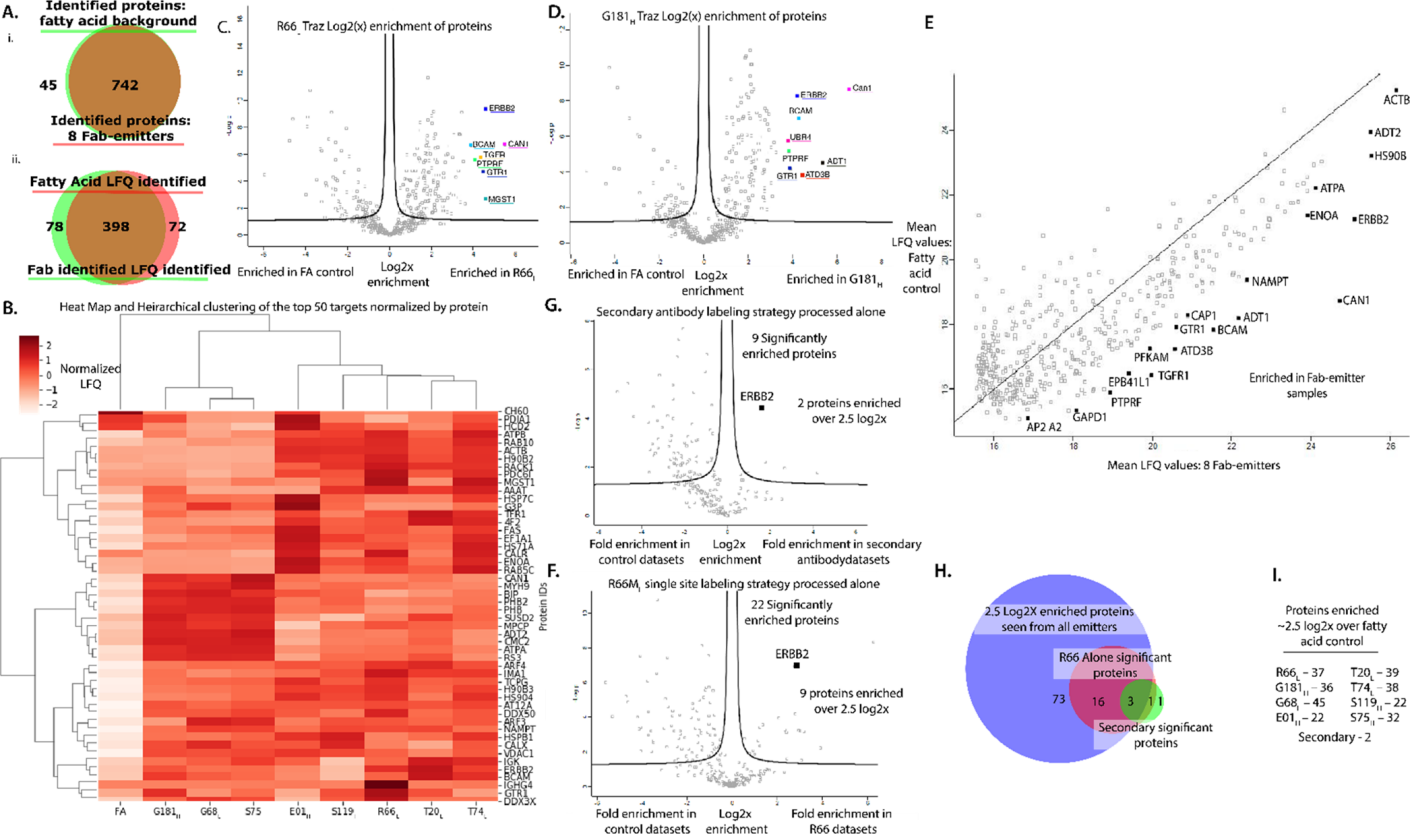
Proteins identified on SKBR3 cells for the eight Fab-catalysts and the secondary antibody-catalyst, compared to nonspecific membrane labeling using the FA-catalyst. A.i. Venn diagram showing the overlap for all high confidence identified proteins identified in FA-catalyst and Fab-catalyst experiments. All high confidence proteins identified in the eight Fab-catalysts were detected at some level in the FA-catalyst experiment. ii. Venn diagram showing the overlap of identified proteins with LFQ values. B. Hierarchical clustering and heat map of the top 50 most enriched proteins for the Fab-catalysts (lanes 2–9), relative to FA-catalyst (lane 1). LFQ values for each protein are normalized within the Fab samples. Non-normalized plots are available in Supporting Information Figure 8. C. Volcano plot for R66M_L_ Fab-catalyst labeling processed together with all eight Fab-catalysts. Proteins enriched greater than 4 log2x units over the FA-catalyst are labeled. D. G181M_H_ Fab-catalyst labeling processed identically to the R66M_L_, showing proteins enriched greater than 4 log2x over the FA-catalyst. E. Mean enrichment of the eight Fab-catalysts plotted against the mean FA-catalyst enrichment levels. Proteins most enriched over FA-catalyst background are highlighted across a range of LFQ values. The overall range of LFQ values likely reflects differences in protein abundance. Proteins that fall furthest off the unity line are likely HER2 neighbors. Not surprisingly, the three most highly enriched hits derive from HER2 and the Fab-catalyst light and heavy chains. F. Volcano plot showing fold enrichment for proteins labeled with the secondary antibody binding to Traz, relative to the FA-catalyst. Note there were only nine high-confidence identifications, and only four that were enriched over 2.5 log2x over the FA-catalyst control, including HER2. G. A comparable volcano plot showing fold enrichment for proteins labeled with the R66M_L_ Fab-catalyst, compared against the FA-catalyst control. Note there were 22 high-confidence identifications of which 9 proteins were enriched over 2.5 log2x over the FA control. H. Venn diagram showing overlap of highly enriched proteins observed in all eight Fab-catalysts, compared to those in the R66M_L_-only data set and the secondary antibody-catalyst data set. I. List of eight Fab-catalysts and total number of proteins seen enriched for each by more than 2.5 log2x relative to the FA-control.

We next analyzed labeling from the single-catalyst Fabs for the proteins that were enriched by proximity labeling. Remarkably, there was near complete overlap for the sum of all proteins detected at any level by each of the eight Fab-catalysts and the FA-catalyst (Figure 4Ai and 4Aii). We next quantitatively compared the protein-specific fold enrichment values for each Fab-catalyst’s labeling versus the FA-catalyst control (Figure 4B). Proteins were filtered for those with at least three LFQ values in either one of the Fab-catalyst site mutants or in the FA-catalyst control. Combining data for all eight site-specific Traz Fabs gave higher confidence identifications, with 457 total proteins remaining in the data set after screening. Furthermore, a larger Maxquant run of 48 experiments identified more peptides for LFQ quantification, increasing the LFQ values with greater total ID’s.

We further analyzed the top 50 proteins with the highest enrichment values in the Fab-catalysts, by generating a heat map with hierarchical clustering (Figure 4B). The heat map clearly distinguishes the group of eight Fab-catalysts from the FA-catalyst. In detail, each Fab-catalyst is somewhat different, likely reflecting the different sites for the Ir-catalyst. We also present enrichment differences between Fab-catalyst and FA-catalysts by classic volcano plots (Supporting Information Figure 7). We combined the data sets for the Fab-catalysts to increase the statistical confidence of targets. This allowed less enriched hits to emerge relative to the FA-catalyst control. Figure 4C,D shows example volcano plots for two of the Fab-catalysts, R66M_L_ and G181M_H_, which are furthest from each other on the Fab and show the greatest spread of differences.

Mean LFQ values from all Fab-catalysts were graphed against mean LFQ values for each FA-catalyst to determine enrichment (Figure 4E). Results demonstrate that across the entire range of LFQ values, specific proteins were selectively enriched by the Fab-catalysts against FA-control. The proteins labeled are those that are substantially and consistently enriched among the eight Fab-catalysts. As expected, ERBB2 (HER2) and the primary antibody light and heavy chains from the Fab-catalysts are among the most abundant and enriched proteins.

We then sought to compare our site-specific labeled primary Fab-catalyst to the nonspecific labeled secondary antibody approach described in μMap. We produced a comparable secondary antibody catalyst probe labeled with roughly six to eight catalysts, which was then added to cells prebound with Traz. Samples were analyzed in biological triplicate and technical duplicate and processed using the same methods described for the Fab-catalysts (Supporting Information Table 3). In total, the secondary antibody catalyst detected 334 proteins (<1% FDR), with nine proteins significantly enriched over background and only five seen >2.5 log2x enriched including the HER2 and the Traz antibody (Figure 4F; Supporting Information Figure 6). By comparison, we found 16 proteins enriched in the R66M_L_ Fab-catalyst by >2.5 log2x, including virtually all those found in the targeted secondary antibody catalyst experiment (Figure 4H). These data indicate that site-specific catalyst attachment can provide higher resolution on cells than the multisite NHS labeled secondary antibody catalyst.

Comparing volcano plots for all eight of the Fab-catalysts (Supporting Information Figure 7), we see each Fab-catalyst has between 20 to 40 proteins enriched over 2.5 log2x, relative to the FA-catalyst (Figure 4H). A Venn diagram of these highly enriched proteins shows each Fab-catalyst has a unique pattern (Supporting Information Figure 7), but we identify 16 proteins that are commonly labeled by four or more Fab-catalysts (Supporting Information Figure 7).

Of the proteins consistently highly enriched, the majority are known to be secreted or membrane proteins, with 61 of 79 being assigned to the membrane in the GO database (Supporting Information Figure 7D). String analysis shows that 71 of 88 proteins are known to have functional links to HER2 (Supporting Information Figure 6D,E; Supporting Information Table 4). We have further tabulated the common hits with the greatest amount of literature associated with HER2 biology or breast cancer (Table 1).

**Table 1:** Selected Examples of Neighbors, Highlighted for Relevancy to Her2+ Cancer or HER2 Signaling

MS ID	protein name	potential relevance to Her2+ cancer
CAN1	calpain-1	a protease linked to numerous disease states, specifically tumor migration and progression^51^
TGFR1	TGF-beta receptor type-1	a signaling protein linked to inhibition of early cancer progression, however in late cancer progression is associated with increasing tumorigenicity and invasiveness^52^
PTPRF	receptor-type tyrosine-protein phosphatase F	a phosphatase which regulates signaling at cell–cell junctions; overexpression has been implicated in decreased survival in prostate cancer^53^
BCAM	basal cell adhesion molecule	an adhesion protein which through not fully established pathways has been linked to tumor progression, size, and negative survival rates^54^
ENOA	alpha-enolase	a glycolytic enzyme which is known to have a secondary role in cancers as a cell surface receptor increasing tumorgenicity^55^

## Discussion

### Single-Site *In Vitro* Experiments Allow Structure-Based Calibration of the Carbene PLP Labeling Technology at Single Residue Resolution

The recent development of μMap technology has opened exciting opportunities for higher resolution and higher confidence identification of protein partners on cells. In addition to better defining protein complexes *in situ*, we believe the technology has the theoretical potential to help provide structural information for protein complexes *in vitro*, much like spectroscopic methods such as fluorescence energy transfer (FRET) or chemical cross-linking.^28^ Thus, we focused on calibrating labeling distances and residues *in vitro* within the structurally well-defined Traz–HER2 complex using site-specific Ir-catalyst conjugation. We quantified sites of carbene labeling at single amino acid resolution from simple tryptic peptide mixtures without the need for biotin enrichment.

From intramolecular experiments on the site-specific Fab-catalysts, we found that virtually all amino acids can be labeled, as anticipated from the known chemistry of highly reactive carbenes.^42^ However, some systematic variations were context dependent. Such generally broad amino acid coverage allows labeling of many sites, thus increasing the sensitivity of detection. Other PLP methods that generate hydroxy radicals or reactive biotin–AMP are inherently less sensitive, as they react predominantly with tyrosine/tryptophan and lysines, respectively. About 20 highly buried residues were not labeled in the Traz-Fab, including Met and Ile. When correlated with SASA, we found that as long as an amino acid showed some exposure, it could be labeled.

With techniques to measure distances at the molecular scale, such as fluorescence energy transfer (FRET), nuclear Overhauser effects (NOE) using NMR, and paramagnetic probes using EPR,^43−45^ it is critical to empirically calibrate the distance dependence of effects on structurally defined sites with model systems. We applied the same reasoning to DET labeling here using intramolecular labeling of single-site defined Fabs and intermolecular labeling to its HER2 binding partner. Empirically, we see that labeling was rather uniform out to ∼110 Å. However, unlike FRET and NOE, we see considerable noise in the distance dependence of labeling. We hypothesize that this is due to reaction efficiency, differences in how labeled peptides fly in the MS, and incomplete peptide coverage. Nonetheless, the broad and somewhat uniform amino acid coverage, the simplicity of MS analysis, and the availability of recombinant antibodies to many disease targets potentially can provide a useful and orthogonal information to use for structural characterization, even in complex samples.

**Figure 5:**
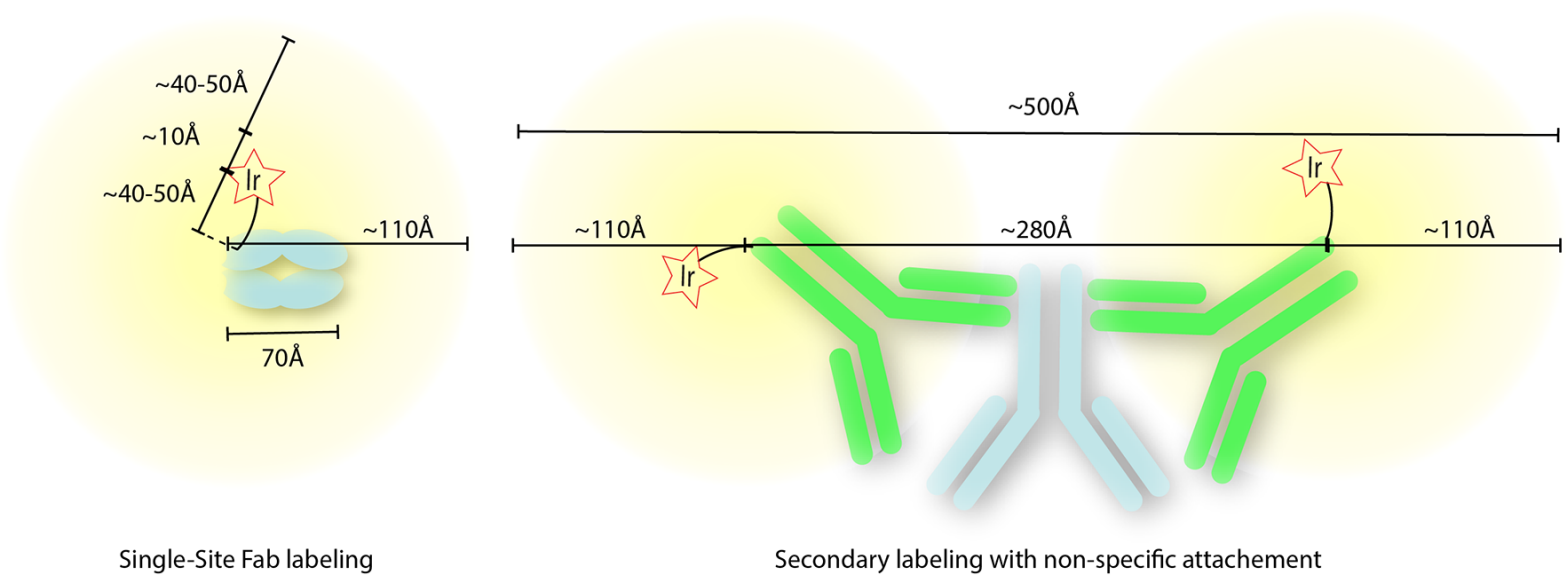
A. A cartoon schematic of the radial labeling distances estimated, compared to experimental measurements. Theoretical distances are represented by the trio of determinants: a ∼10 Å distance for the DET process between the photocatalyst and diazirine, a ∼40–50 Å diffusion limit for carbenes in water, and a ∼40–50 Å leash from the Ir-catalyst to the Cα attachment site on the protein. These roughly match our maximum experimentally derived distances of ∼110 Å. B. A schematic of this model applied to a secondary antibody system with approximate distances. This total distance agrees with the previously observed labeling distance of this complex of 540 ± 120 Å.

The DET process involves an electron transfer from the triplet excited state on the Ir-catalyst to the strained three-member ring of the diazirine within about 10 Å.^46^ Once the carbene is triggered, it can diffuse around 40 Å^47^ before it is quenched by water (given the half-life of the carbene in water of 7 ns). In our system, the Ir-catalyst is on a ∼50 Å leash from the Cα of the Met on the Fab-catalyst (estimated by Avogadro^48^). By summing the distance, diffusion, and leash length, we would theoretically expect a labeling distance radius that is strongest at approximately 70 Å and a possible extreme distance of around 110 Å, which closely matches our measurements (Figure 5). Additional factors not captured here could include the dynamics of the leash and the protein, the potential binding of the Ir-chelate as it surveys the protein surface, and finally exclusion from penetrating the protein itself. All these factors should affect sites that are labeled, which is why it is critical to empirically determine distance relationships. Previous estimates using stimulated-emission depletion microscopy (STED)^27^ and three different constructs provided very useful distance ranges for biotin–carbene labeling for the μMap format^30^ of 570 ±120 Å, 540 ± 120 Å, and 570± 120 Å. If we overlay the estimates we have made upon the primary and secondary antibody format, the system used in Oakely et al.,^27^ which used a streptavidin–fluorophore conjugate for detection, we can rationalize the ∼550 Å range reported. They also noted that the large system would extend the range of the carbene labeling due to the size of these antibody–target–streptavidin complexes.

### On-Cell Labeling with Single-Site Fab-Catalysts Revealed New Members of Signaling Active HER2 Neighborhoods with Higher Resolution and Higher Confidence

The Fab derived from Traz is a small protein (∼50 kDa) and does not perturb signaling. This allowed us to probe the active HER2 neighborhood in HER2-overexpressing cells, such as SKBR3 cells. We sought to establish whether single-site catalyst Fabs would perform better with a smaller radius of labeling compared to the μMap method, in terms of number and confidence of identifications and sensitivity for high and low abundance partners. To determine the specificity of labeling, we hypothesized that a nonspecific FA-tethered catalyst would more accurately sample the membrane protein background.

Membrane tethering of enzymes has been shown to dramatically improve the depth of engaging membrane proteins by a sialidase,^49^ a peroxidase,^50^ or subtiligase,^12^ by more than 20-fold. Thus, we created a general membrane-anchored catalyst probe through attachment of the Ir-catalyst to a metabolically incorporated FA-catalyst. The FA-catalyst produced comparable and even slightly higher numbers of identifications compared to traditional CSC methods for surfaceomics^8^ (between 600 and 1000 membrane proteins in a typical data set). By comparing the Fab-catalysts to the FA-catalyst, we identified about two dozen highly enriched partners (>2.5 log2x) with high confidence for the Fab-catalyst probe (R66M_L_), while only four were labeled when the photocatalyst was attached to using the secondary antibody μMap. We believe this reflects closer labeling from a single-site primary Fab-catalysts that is positioned much closer to the HER2 target. The Fab-catalyst and FA-catalyst labeled largely the same ∼800 proteins on the cell, though significantly differing in LFQ values as seen by the enrichment values and volcano plots. Bringing the photocatalyst directly against the target allowed better enrichment of the partners that interact with HER2.

All eight of the Fab-catalysts produced somewhat unique collections of two to three dozen high-confidence hits reflecting their different vantage points in labeling the neighborhoods. Interestingly, we find different locations of the Ir-emitter yield overlapping proteins, but with some differences in the highly enriched neighbors. Not surprisingly the data suggest different positions of the emitter on the antibody can affect the specific observed neighbors. We doubt that all these proteins form one large complex with HER2, but likely reflect a number of complexes. Most of these have not been reported to bind with HER2 directly, but the majority have functional links to HER2 by STRING analysis. To highlight examples of neighbors that can be identified we list a common set of neighboring proteins identified from the eight catalyst sites, with known functional links to HER2 as shown in a STRING analysis (Table 1, SI Table 4). These candidate interacting proteins will require more detailed cellular experiments to validate a structural and functional role with HER2.

## Conclusions

The single-site Fab-catalyst probes help to calibrate the distance resolution of carbene labeling. Our studies demonstrate the broad breadth of residue-specific labeling and validate increased resolution and sensitivity for site-specific attachment on the primary antibody. It is relatively simple to create single-site catalysts to other binding proteins, which may be useful for assisting in characterizing structures and stoichiometries of protein complexes in vitro and on cells. Single-site catalyst probes allow one site to label surrounding proteins in a distance-restricted fashion. In addition, it should be possible to increase and decrease the resolution by using catalysts with shorter or longer leashes, or by switching out the reactive intermediate used. For example, recently it has been shown^27^ that photocatalytic triggering of different phenyl azides to generate reactive nitrenes in μMap format increased labeling from 700 to1200 Å, and with phenols up to ∼3000 Å. Given the abundance of recombinant proteins and antibodies, we believe single-site conjugated binding proteins, coupled with nonspecific FA-catalyst controls, will increase the confidence and spatial resolution for transient protein interactomics.

## Supporting Information Available

A detailed methods section is available in the Supporting Information, as well as all of the supplemental figures and tables referenced in the manuscript. Multiple files are separate excel documents with raw data from the studies. The Supporting Information is available free of charge at https://pubs.acs.org/doi/10.1021/acscentsci.3c01473.

Table S3 spreadsheet containing raw data from the studies (XLSX)
Table S4 spreadsheet containing raw data from the studies (XLSX)
A detailed methods section as well as all of the supplemental figures and tables referenced in the manuscript (PDF)
