# Supplementary material for: Photoproximity Labeling from Single Catalyst Sites Allows Calibration and Increased Resolution for Carbene Labeling of Protein Partners In Vitro and on Cells
Source: ACS Cent Sci. 2023 Dec 26;10(1):199–208. doi: 10.1021/acscentsci.3c01473 (PMC10823516; doi:10.1021/acscentsci.3c01473)
Supplement: Supplementary file 3 — oc3c01473_si_003.pdf [file oc3c01473_si_003.pdf]

Photo-proximity labeling from single catalyst sites allows calibration and increased resolution for carbene labeling of protein partners *in vitro* and on cells

Authors & Affiliations:

Thomas G. Bartholow[1], Paul W.W. Burroughs[1], Susanna K. Elledge[1,2], James R. Byrnes[1,3], Lisa L. Kirkemo[1,4], Virginia Garda[1], Kevin K. Leung[1], James A. Wells[1,5*]

[1]*Department of Pharmaceutical Chemistry, University of California San Francisco, San Francisco, California, 94158, USA.*

[2]*Present address: MIT, Cambridge, MA 02139*

[3]*Present address: Interline Therapeutics, Brisbane, CA 94005*

[4]*Present address: 3T Therapeutics, S. San Francisco, CA 94128*

[5]*Department of Cellular & Molecular Pharmacology, University of California San Francisco, San Francisco, California, 94158, USA.*

[*]*Corresponding Author: jim.wells@ucsf.edu*

Contents

Supplemental Methods

SI Figure 1. Methionine sites and conjugation chemistry used for step-wise addition of Ir-catalyst to the Fab of Traz.

SI Figure 2. Intramolecular labeling of Traz by the Ir-catalyst from eight different Met sites.

SI Figure 3. Carbene labeling of the HER2- Traz complex from each of the Traz Ir-catalyst conjugates

SI Figure 4. Distance dependence for the labeling of the same residue on HER2 from each of the eight catalyst sites on Traz.

SI Figure 5. Comparing the labeling of R66M_L alone and the secondary labeling strategy relative to the most enriched proteins from all eight Traz catalysts.

SI Figure 6. Biological details and overlap of high confidence identified neighbors.

SI Figure 7. Volcano plots for each of the eight Traz catalysts used in the study.

SI Figure 8. Comparing the LFQ values from CSC, FA azide, and unbound Ir-secondary controls.

SI Table 1. Performance of the three controls chosen for comparison.

SI Table 2. Mean LFQ values from experiments.

SI Table 3. Log2x transformed LFQ values for each replicate and emitter site.

SI Table 4. STRING analysis values.

Supplemental Methods

Traz (Traz) Fab labeling protocol

Single site mutated Traz fab's were utilized from a previous study[32].The fab mutants were grown following a previously established protocol[1]. DE3 cells were grown in supplemented Terrific Broth at 37° C for 6 hours, followed by 18 hours of growth at 30°C. After growth cells were lysed on a Qsonica sonicator in PBS and purified by protein A affinity chromatography using VWR Hitrap protein A 1mL columns. After purification proteins were verified by SDS-Page gel and concentrated for oxaziridine reactions. Reactions were performed following protocols published in Elledge. Et al, 2020[2]. Briefly, proteins were reacted at room temperature on the bench at 10µM antibody with 100µM oxaziridine in PBS. Labeling was allowed to occur for one hour before verifying labeling by Waters Xevo UPLC-MS. After oxaziridine labeling, proteins were buffer exchanged by Zeba spin column before loading with DBCO-Ir probe. Labeling was performed with 5-fold molar excess probe overnight at 4° C before buffer exchange by Zeba column and confirming labeling by Mass spectrometry. Estimates of Iridium probe size were performed by building the catalyst in Avogadro[3] and finding the maximum possible distance given stable geometries.

In vitro DET labeling experiments

DET labeling experiments were performed *in vitro* by mixing 9µM Traz-catalyst with 100µM diazirine-biotin. Blue light illumination was performed on a PennPhD Photoreactor M2 for 10 minutes at full power. After illumination samples were flash frozen for later thawing and processing. Preparation for Mass Spectrometry was done using a Preomics Ist kit. Procedures were followed as described in the user manual with the modification of lysis by only a half volume of Trypsin/LysC for half an hour on the bench, to avoid excess protease activity on the samples. Samples were dried on a Genevac EZ-2 centrifugal evaporator before resuspension and normalizing to a 200ng/µL concentration. 1µL of samples was run on a Bruker TimsTOF. Samples were run on a NanoViper system with a Bruker TEN 10cmx75µm nanoflow column. *In vitro* samples were run on a 45 minute gradient from 4-32% solvent B (Solvent A: 2% acetonitrile with 0.1% TFA Solvent B: Acetonitrile with 0.1% TFA). *In vitro* analysis was performed on the PEAKs[4] server using a 1% FDR and searching for a 616.25424 diazirine biotin label. A 20 PPM precursor mass error tolerance and 0.1 Da fragment mass error was used in analysis.

Labeled residues were collected and analyzed based on the number of instances of labeling. First analyzing by distance of labeling vs angstrom distance from the Ir-catalyst bearing residue. All distances were calculated from the PDB 1N8Z[5] and residue identities were taken from the PDB file. Analysis was performed in BioPython[6,7]. The second round of *in vitro* experiments was performed with equimolar concentrations of Her2 and catalyst-Traz. For consistency a 9µM antibody-catalyst was used along with 9µM Her2 and 100uM diazirine-biotin. Preparation for Mass Spectromety was then performed identically to the previous samples. For quantification the Mass Spectrometry .Mzml files were downloaded and reanalyzed in Skyline[8]. In Skyline analysis was performed using the same FDR and settings as Peaks. Skyline quantification was performed with a linear regression fit and normalizing to the total ion count. Results were exported from Skyline with the calculated quantification values,

peptide identity, experiment, and isotope dot product. Quantified peptides were filtered by an isotope dot product of 0.85 or greater. In the "Her2 protection" analysis residues within 7.5 angstroms were identified from the 1N8Z crystal structure and collected. Residues within 10 angstroms were identified by collecting residues within 10 angstroms and removing any of the residues identified within 7.5 angstroms to find the unique residues. The same was done for 15 angstroms distance. Then quantification was performed by summing all the quantified labeling at any residue within each unique distance group.

On Cell labeling protocol

On cell labeling was performed with SKBR3 cells obtained from the UCSF cell culture facility. Cells were grown in T-175 flasks with RPMI media supplemented with 10% FBS and 1% penicillin/streptomycin. Cells were grown up to 70-80% confluency before harvesting. Cell harvesting was performed by first washing flasks with PBS before scraping cells into PBS. Cells were checked for count and viability, ensuring that they were at least ~80% live before aliquoting and storing on ice. In all on cell experiments 5 million cells were used. After scraping and washing, cells were collected and antibodies for labeling were added at 5µg and incubated on a rotator at 4°C for 30 minutes for binding.

Single site Fab labeling was performed by immediately incubating the cells with 5µg fab and allowing 30 minutes for binding on a rotator at 4° C. Following Fab binding 3 rounds of PBS washes were performed to wash the cells and avoid unbound antibody. After washing the cells were brought up in 1mL of 100µM diazirine biotin in PBS and irradiated with blue light for 10 minutes. After irradiation the cells were washed 3 final times with PBS to avoid residual diazirine in the samples then flash frozen for later processing. In the case of Secondary catalyst labeling the same procedure was followed, with the exception of two antibody binding sets. First a Traz-IgG (Obtained from Selleck chemicals catalog #A2007) antibody was bound for 30 minutes at 4° C on a rotator. Followed by washing then binding of the Ir catalyst conjugated secondary for another 30 minutes. The Ir secondary (ThermoFisher catalog # 12-4998-82) was prepared by the same NHS labeling protocol used in the micromap protocol for consistency[9]. After labeling, cells were washed identically to the Fab samples and stored.

Biotinylated proteins were first prepared for mass spectrometry by resuspension of the frozen cells into 2X RIPA buffer with added Halt ThermoScientific protease inhibitor cocktail and 5mM EDTA. Cells were lysed on a by sonicator and after incubated in the RIPA buffer for 10 minutes on ice. After they were centrifuged for 10 minutes at 21,000g to pellet cellular debris. The supernatant was collected and batch bound to Thermo Scientific High capacity streptavidin-agarose beads for 2 hours at 4° C while on a rotator. After the beads were washed on Biorad Bio-spin small volume chromatography columns using vacuum to wash 3 solutions over the beads. 5mL of 1X RIPA with 1mM EDTA was flowed over beads, taking care to disrupt the bead bed during washing. Followed by 5mL of PBS with 1M NaCl and finally 5mL 50mM ammonium bicarbonate with 2M urea again taking care to disrupt the beads in order to ensure full washing. These washed beads were then processed for mass spectrometry using the Preomics Ist kit. After preparation the peptides were dried on a SP Genevac before bringing up in 2% acetonitrile and .1% TFA in Mass Spectrometry grade water and diluting all samples to 200ng/uL. All samples were injected twice onto a Bruker TimsTOF. Samples were run on a NanoViper system with a Bruker TEN 10cmx75µm nanoflow column. *In vitro* samples were run on a 90 minute gradient from 4-32% solvent B (Solvent A: 2% acetonitrile with 0.1% TFA Solvent B: Acetonitrile with 0.1% TFA) This procedure was used for all cellular samples.

Fatty acid catalyst experiments were performed by lipid starving the cells for 1 hour by removing media, washing once with PBS, then replacing media with RPMI with 1% Pen/strep but no supplemented FBS. After lipid starvation the media was removed and Azido fatty acid media was added. To prep the Azido caproyl acid a 100uM solution in DMSO was saponified with 20% molar equivalent potassium hydroxide and by heating to 65°C for 15 minutes. A 100uM solution of fatty acid in RPMI was warmed to 37°C before addition to the lipid starved cells. Cells were allowed an additional hour to grow in the lipid supplemented media. After the final incubation cells were washed twice in PBS and scraped by the same protocol as with Traz labeled cells. After scraping cells were washed once in PBS and then reacted for 1 hour at 37°C with 100uM DBCO-Ir probe. After the reaction cells were washed 3x with PBS to remove background and brought into PBS with 100uM Diazirine-biotin. Cells were blue light irradiated for 10 minutes before washing twice with PBS to remove unreacted diazirine and freezing. Cells were then processed identically to the previous samples. WGA-HRP CSC experiments were performed as described in publication[49]. The same T-175 flasks were used for collecting cells. Samples were processed identically to all previous cellular labeling samples. In the controls using a Ir-catalyst secondary with no primary the same procedure as catalyst-Fabs was used. Briefly after collecting cells they were incubated for 30 minutes at 4°C to allow for binding. After cells were washed twice before resuspending in 100µM diazirine biotin and labeling with blue light. After washing the samples post blue light they were frozen for preparation.

Proteomics sample processing

Proteomics samples were processed in Maxquant 2.0.3.0[10,11]. Peptide search was performed with the SwissProt GOCC Plasma Membrane database. A FDR rate of 0.01 was used for searches, LFQ values were obtained from Maxquant using their classic normalization. After peptide identification additional filtering was performed in Perseus[12,13]. First peptides were excluded which were identified by a single site, identified as reverse peptides, or peptides identified by Maxquant as likely contaminants. Next proteins were eliminated which were identified by only one unique peptide. Experiments were grouped together by labeling site. Following this, proteins were Log2(x) transformed and proteins were eliminated which were not identified by LFQ in 3 replicates of at least one experimental set. Imputation was performed with a down shift of 2.2 and sampling a width of 0.3 using the Perseus "Replace missing values from normal distribution" command. Following this Volcano plots were created between groups with significance tested by a t-test, using 250 randomizations with a FDR of 0.05 and S0 of 0.1. In presented plots IGK was removed as it originated from the antibody used in the experiment. Hierarchical clustering was performed by averaging groups by geometric mean in Perseus. Clustering was performed in Python using the Seaborn statistical data visualization package[14], an unweighted pair group method with arithmetic mean was used with a Euclidian distance metric. Proteins highlighted for further literature search were chosen by taking a collection of the most enriched proteins (Over 4x log2x enriched) from the 8 emitter sites. All of the proteins were indicated to interact with ERBB2's network by STRING analysis. The full list of proteins meeting this level is: CAN1, MPP6, ENOA, MGST1, ANXA1, BCAM, TGFR1, PTPRF, GTR1, ADT1, EPHB3, MPZL1, ATD3B, ST14, UBR4, LSR, PLXB2, KHDR1, ERBB2, and IGK. A literature search revealed interesting roles for each of these proteins but the final 5 chosen for presentation in the main text were those with a diverse set of possible roles.

All protein visualizations were performed in Pymol.

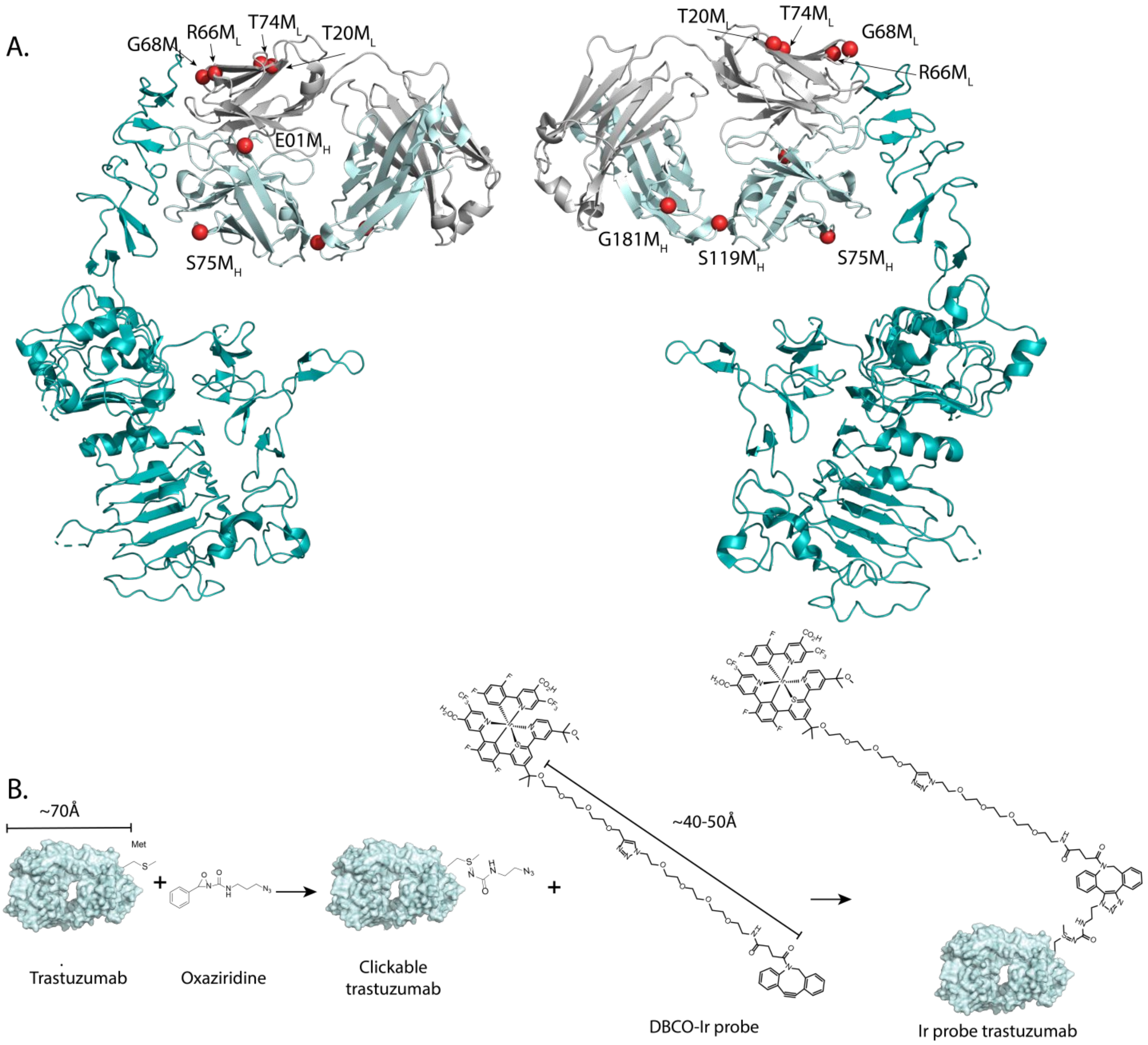

SI Figure 1. Methionine sites and conjugation chemistry used for step-wise addition of Ir-catalyst to the Fab of Traz. **A.** Ribbon diagram showing front and back view of the complex between the extracellular domain of HER2 bound with Traz. The eight sites across light and heavy chains in Traz that were mutated to methionine in order to perform site-specific oxaziridine conjugation are shown in red dots distributed. The HER2 structure is shown in Teal with the Traz light chain in gray and heavy chain in light cyan **B.** The two reaction steps involved to create the DET-probe conjugated to Traz are shown (i) oxaziridine conjugation chemistry onto the Met to attach the azide and (ii) attachment of the Ir catalyst using DBCO click chemistry. The protein is minimized in scale in order to highlight the structure of the probe.

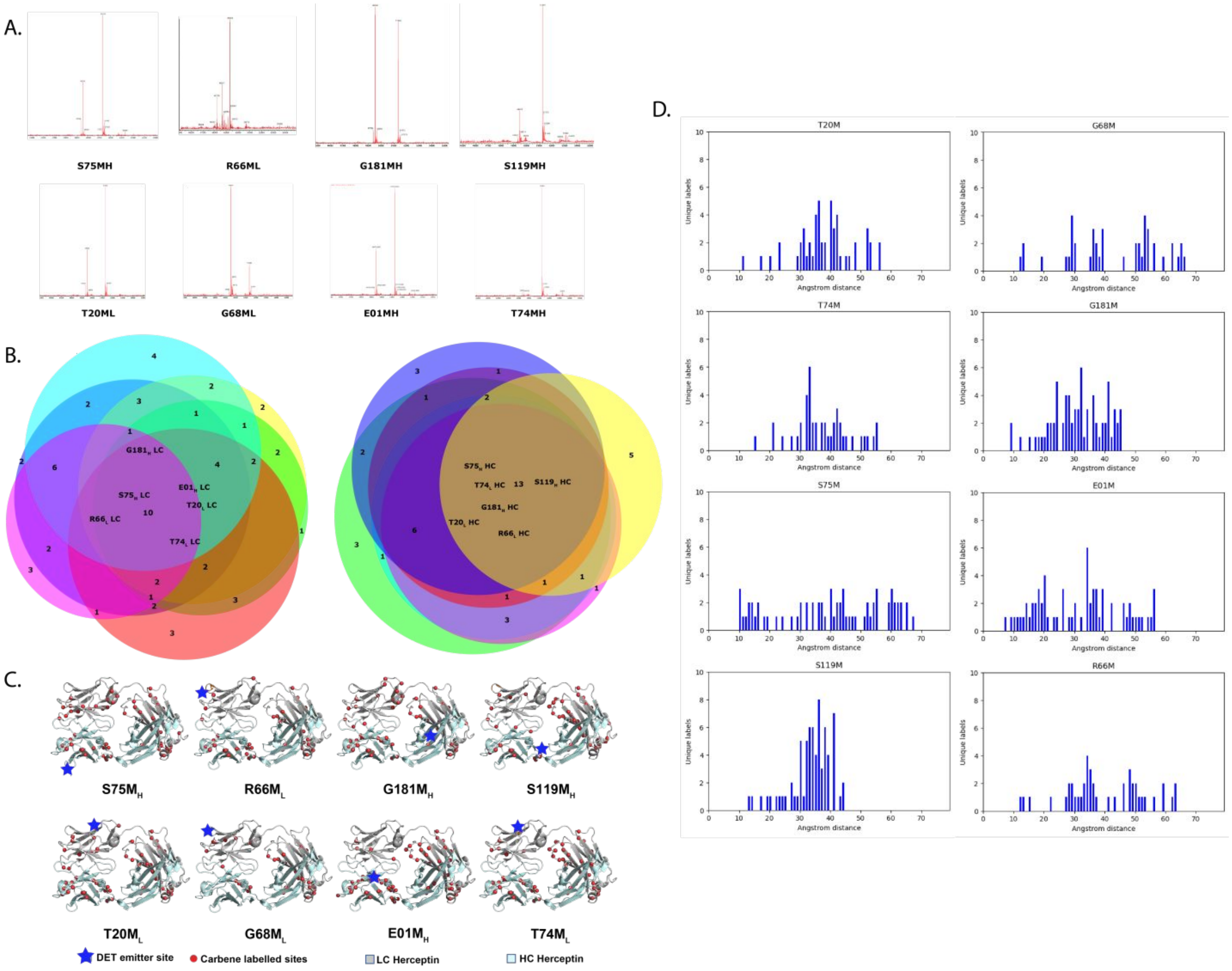

SI Figure 2. Intramolecular labeling of Traz by the Ir-catalyst from eight different Met sites. **A.** MS traces of the Ir-catalyst conjugated Fabs. 100% labeling was not achieved in all cases but was not found to be essential to experiments. **B.** Venn diagram of the labeling of for six of the eight catalyst sites on the Traz light and heavy chain (Light cyan and grey ribbons, respectively). Full reports of labeled sites are available in the supplemental tables. Most sites overlap but each catalyst has a unique detailed labeling pattern. **C.** Sites of carbene insertion (red dots) from each of the eight catalyst sites (shown by star). D. Individual plots of the intramolecular labeling of each of the 8 emitter conjugated Trazs. Labels are identified from experiments without Her2 binding.

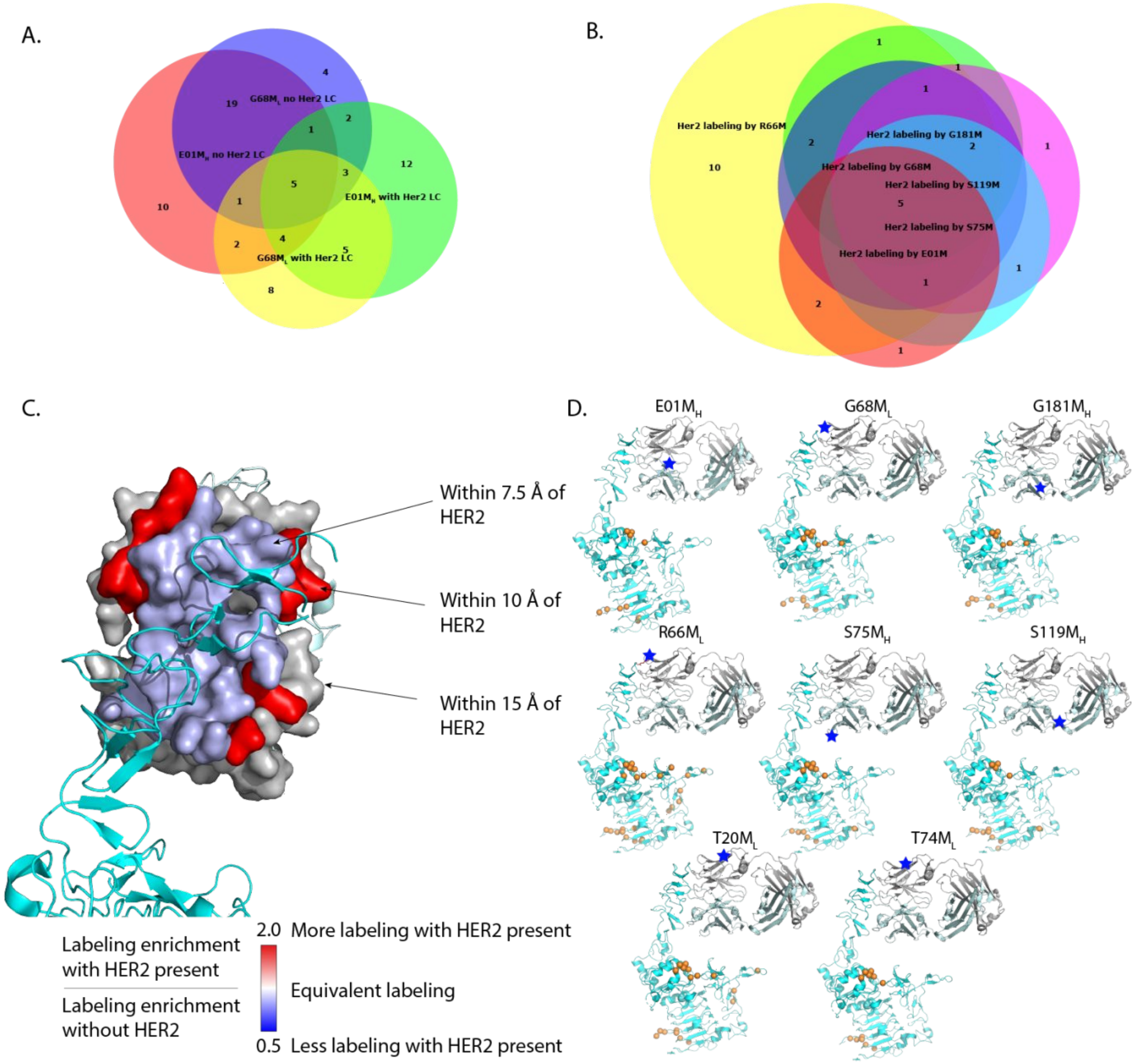

SI Figure 3. Carbene labeling of the HER2- Traz complex from each of the Traz Ir-catalyst conjugates **A.** Venn diagram of labeling comparing two emitter sites with and without HER2. Demonstrating that addition of the partner appears to have effects on where labels are seen. **B.** A Venn diagram of labeling by 6 of the 8 Fab-catalysts. Many of the carbene labeling sites are in common on HER2 although the closest one, R66M$_L$, systematically had more than the others. **C.** Assessment of the protection by HER2 of the emitter-Fab self-labeling. The contact surface of the emitter-Fab was rendered in space filling view and colored based on the ratio of labeling in the presence and absence of HER2 (teal ribbon). Ratios are generated by combining all the labeling seen for residues at the described distance from HER2. **D.** The sites of carbene labels (organ spheres on alpha carbons) seen from each of the eight catalysts. The HER2 structure is shown in Teal with the Traz light chain in gray and heavy chain in light cyan. Emitter sites are highlighted as blue stars.

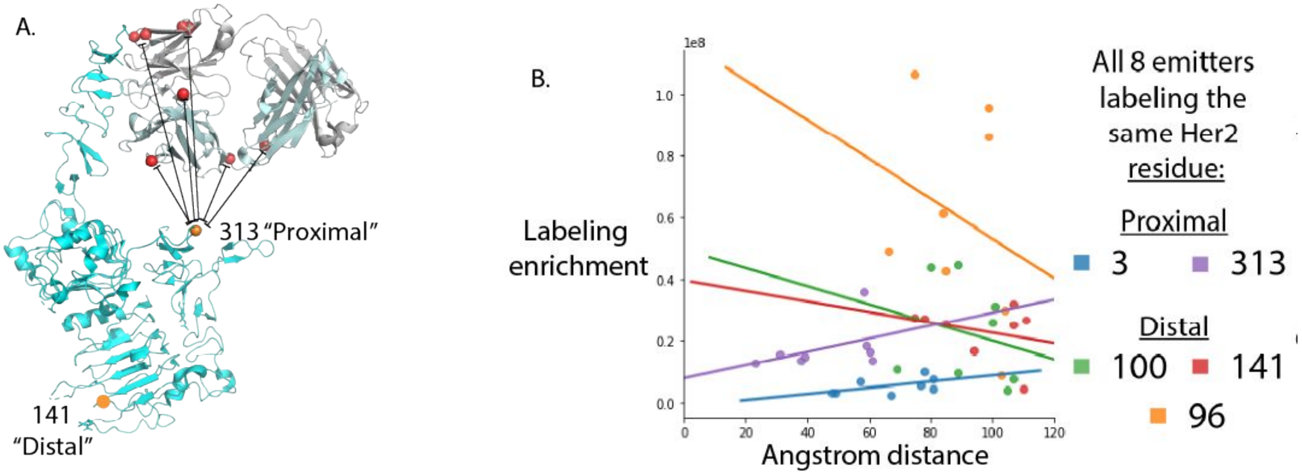

SI Figure 4. Distance dependence for the labeling of the same residue on HER2 from each of the eight catalyst sites on Traz. **A.** Structure of Traz (light cyan and grey ribbons) bound to HER2 (teal ribbons). Traz catalyst sites are indicated in red dots and example common proximal or distal residues on HER2 labeled by the carbene indicated in orange dots. **B.** Distance dependence for carbene labeling at five common residues on HER2 from the eight different catalysts on Traz. Note that proximal residues show relatively flat even profiles across distance while those distal show a loss at greater distance dependence and both terminate near 110Å.

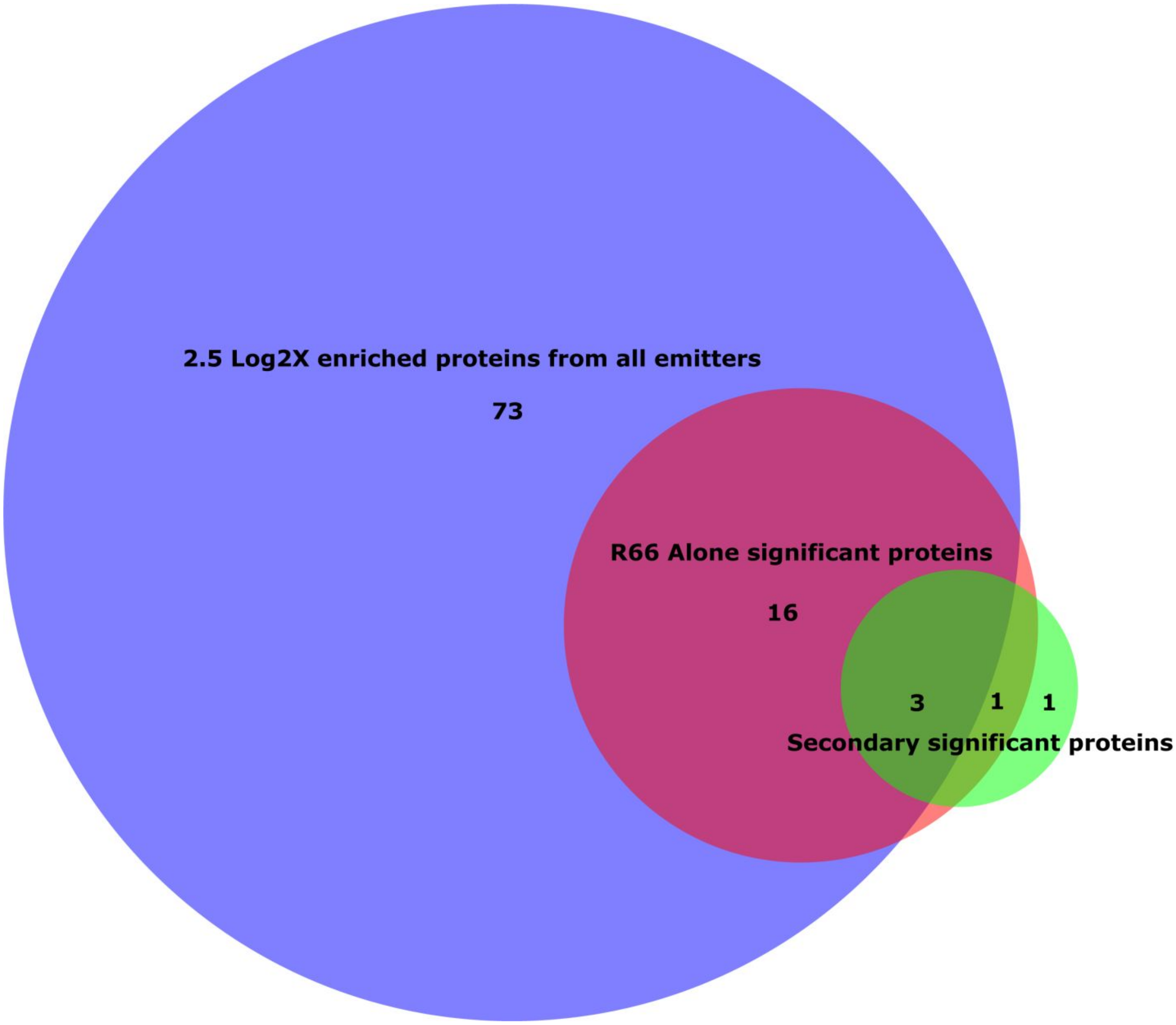

SI Figure 5. Comparing the labeling of R66M_L alone and the secondary labeling strategy relative to the most enriched proteins from all eight Traz catalysts. The vast majority of the proteins identified by R66M_L alone and most of the secondary emitter significantly enriched proteins are the same as those seen in the larger dataset.

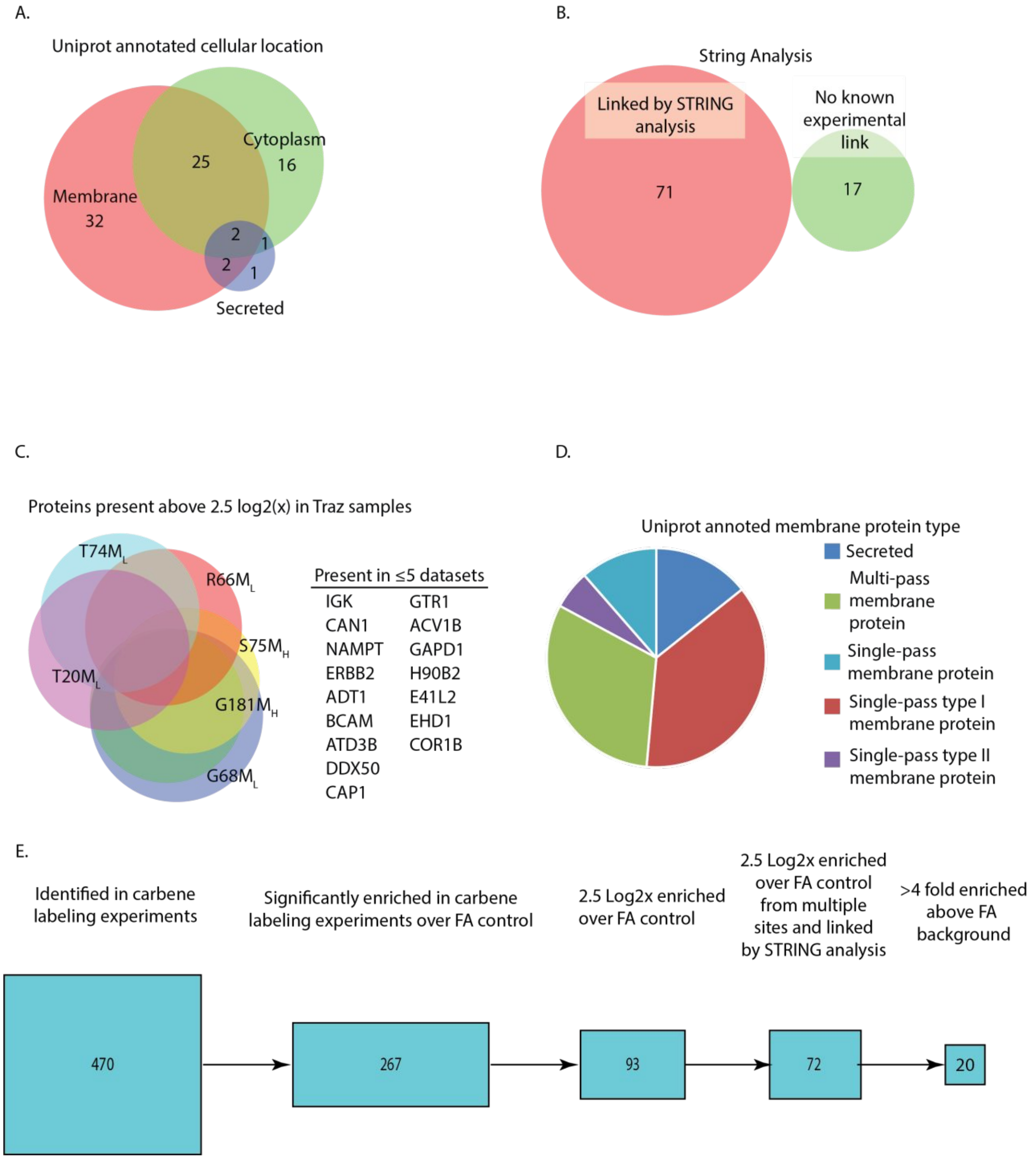

SI Figure 6. Biological details and overlap of high confidence identified neighbors. A. A Venn diagram showing the overlap for all high confidence identified proteins identified in the FA-emitter and the Fab-emitters. Virtually all of the high confidence proteins identified in the 8 Fab-emitters were detected in the FA-emitter experiment. B. A String analysis for eachof the highly enriched proteins from the Fab-emitter volcano plots. There was a strong association with HER2 as 71 of 88 proteins have a known link. C. A Venn diagram showing there is considerable overlap among highly enriched proteins from the Fab-emitters and a list of proteins present in at least four of the Fab-emitters. D. The cellular location GO annotation of the highly enriched proteins demonstrating that hits are predominantly identified as membrane or extracellular, with only 16 annotated as cytoplasmic E. A breakdown of the strategy used to narrow identified proteins down to high confidence potential targets.

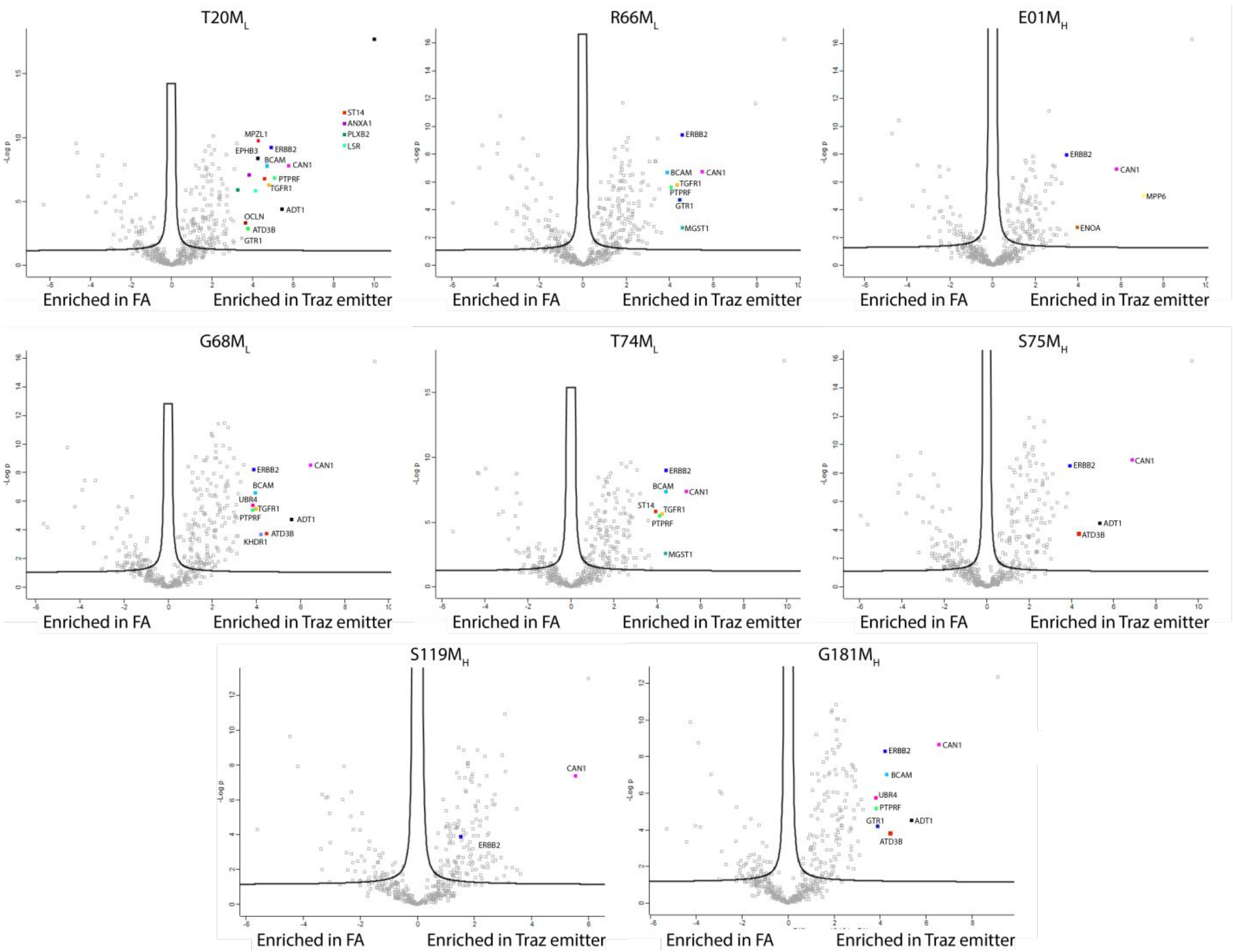

SI Figure 7. Volcano plots for each of the eight Traz catalysts used in the study. The most enriched and common proteins in each of the plots are labelled.

The proteins chosen were

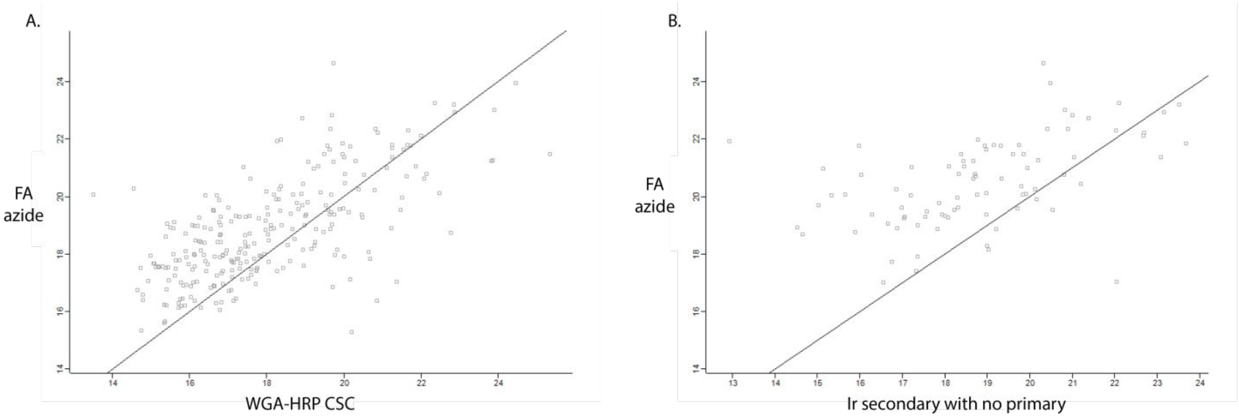

SI Figure 8. Comparing the LFQ values from CSC, FA azide, and unbound Ir secondary controls. A. The mean LFQ of the FA azide and WGA-HRP controls. The line is set to represent even LFQ values. B. The mean LFQ of the FA azide and Ir secondary without a primary control. The line is set to represent identical LFQ values.

SI Table 1. Performance of the three controls chosen for comparison.

Control	Proteins identified	Mean standard deviation (Log2x)
WGA-HRP	543	0.39
Fatty acid azide	787	0.32
Isotype	150	0.67

SI Table 2. Mean LFQ values from experiments. Fab-catalyst sites are grouped together with means calculated for the 3 biological replicates and 2 technical replicates. The fatty acid catalyst experimental group is shortened to FA. The secondary-catalyst experiment is shortened as 2nd.

PROTEIN IDS	$G181M_H$	$G68M_L$	$S75M_H$	$E01M_H$	$R66M_L$	$S119M_H$	$T20M_L$	$T74M_L$	FA	2ND
ENPL	22.806	22.871	22.868	23.269	22.752	22.916	22.657	23.030	22.843	21.419
PLEC	22.140	22.115	22.067	21.846	22.180	22.224	22.104	22.046	21.934	18.776
RB39A	21.386	20.331	21.379	23.222	21.933	21.935	23.198	23.254	20.853	19.253
HNRPF	22.234	22.367	22.256	21.986	21.884	22.325	21.949	21.866	21.806	20.309
EPIPL	22.470	22.437	22.616	20.774	21.695	21.616	21.907	21.373	20.982	19.237
HM13	20.227	21.157	21.131	21.660	21.127	20.663	21.034	21.417	20.739	19.771
RAB15	20.812	20.714	21.049	20.232	20.942	19.633	20.813	19.772	19.669	19.046
GBB2	19.917	20.817	20.768	20.788	20.840	19.837	20.734	20.955	20.036	16.945
PCAT1	19.884	20.810	20.840	20.031	20.636	20.575	20.884	20.536	20.232	15.645
PKP3	20.557	20.544	20.347	19.190	20.105	20.166	20.387	19.805	18.884	17.140
RP1BL	19.727	19.873	19.654	19.819	19.938	20.046	19.803	20.162	19.303	17.600
SURF4	19.822	20.523	19.992	18.448	19.918	20.562	19.902	20.304	19.442	14.597
ARF5	20.754	19.874	20.048	19.638	19.697	20.792	20.574	20.701	18.565	15.292
CTNB1	18.569	18.829	18.725	19.087	19.605	19.191	19.652	19.375	18.694	18.075
DESP	19.256	19.088	19.070	19.304	19.412	19.509	19.484	19.185	18.926	18.491
MIRO2	19.684	19.070	18.115	17.029	18.679	17.962	18.808	17.949	17.438	15.139
CAN2	18.776	18.915	18.880	17.853	18.644	18.616	18.744	18.146	16.929	14.718
DC1L1	18.147	17.650	17.276	17.635	18.442	17.926	18.726	18.376	16.909	16.101
L2GL2	17.516	17.388	16.431	17.393	18.429	18.562	18.112	16.799	17.423	14.676
AT1B3	18.827	20.515	19.717	18.680	18.424	18.657	18.969	19.842	17.401	18.795
G6PI	17.501	17.391	15.736	18.610	18.377	17.689	17.705	18.658	18.291	18.274
HMOX2	18.588	18.456	17.845	18.902	18.287	17.426	18.950	19.004	19.025	17.095
ASPH	19.465	19.594	18.799	17.726	18.214	18.365	18.240	17.851	18.605	15.045
RAB8B	16.833	18.185	19.090	17.309	18.057	17.910	17.793	18.720	18.699	17.508
PREX1	16.898	16.571	16.902	16.326	17.986	16.883	16.629	17.291	16.282	14.399
CNNM4	17.263	16.734	16.452	17.883	17.867	16.561	18.762	17.224	17.311	15.232
TLN2	17.953	18.362	17.663	18.303	17.814	18.376	18.087	18.398	16.894	16.313
SCRB1	15.679	15.709	15.884	17.752	17.812	15.626	18.695	17.587	18.695	22.586
ATPO	18.627	19.650	18.533	19.129	17.788	18.464	17.032	18.064	19.552	14.522
MOT4	18.664	17.916	15.667	18.083	17.729	18.810	18.523	16.975	17.193	14.626
NDUS7	17.443	19.044	17.745	15.950	17.695	17.595	18.407	16.226	16.779	14.637
CBPD	17.440	17.717	16.450	18.247	17.635	18.021	17.075	18.453	17.470	14.652
HIP1R	15.710	15.877	16.320	16.613	17.354	17.333	18.645	16.493	15.556	14.752
NDC1	17.345	15.532	16.339	16.024	17.325	16.630	16.223	16.504	15.915	14.648
FAD1	18.367	18.127	17.083	17.145	17.300	17.677	17.516	17.434	18.430	14.942
RAB31	16.741	16.258	15.759	16.644	17.265	17.075	17.871	17.093	17.180	15.077

**SYMPK**	15.983	17.022	16.282	16.079	17.235	16.815	16.769	16.524	16.840	14.517
**ARFG2**	16.404	16.294	15.861	17.200	17.234	16.178	17.243	16.962	16.130	14.268
**KPBB**	16.518	17.458	17.525	16.447	17.211	16.009	17.369	16.253	16.292	14.406
**RHEB**	16.606	17.176	17.870	15.749	17.146	18.776	17.958	16.779	16.733	14.852
**ITPK1**	16.123	15.731	15.682	16.015	17.108	15.727	15.961	16.524	15.929	14.762
**CUL1**	16.831	16.829	15.954	17.270	17.079	17.247	16.862	17.538	16.464	14.657
**SORT**	16.450	17.385	17.678	17.210	17.038	17.097	17.224	17.543	16.107	14.273
**GNAT3**	17.373	16.739	16.053	16.468	17.031	15.680	16.898	16.457	15.955	14.707
**ERLN2**	17.019	17.859	16.563	17.034	17.026	16.545	17.935	16.633	15.860	14.438
**DSG1**	15.723	15.982	15.802	17.387	17.025	16.495	15.908	15.999	15.666	17.302
**EXOC3**	17.500	16.806	16.817	15.926	16.931	16.381	16.924	16.077	15.658	14.589
**CUL3**	16.179	16.687	16.545	17.813	16.901	16.560	15.885	17.599	15.834	14.753
**AT2B2**	17.689	17.498	16.601	17.817	16.838	17.658	17.542	18.415	16.659	15.526
**OSBP1**	17.091	17.879	17.515	16.801	16.807	17.252	16.031	16.869	15.984	14.433

Supplemental Citations

(1) Hornsby, M., Paduch, M., Miersch, S., Sääf, A., Matsuguchi, T., Lee, B., Wypisniak, K., Doak, A., King, D., Usatyuk, S., et al. (2015) A High Through-put Platform for Recombinant Antibodies to Folded Proteins. *Mol. Cell. Proteomics MCP 14*, 2833–2847.

(2) Elledge, S. K., Tran, H. L., Christian, A. H., Steri, V., Hann, B., Toste, F. D., Chang, C. J., and Wells, J. A. (2020) Systematic identification of engineered methionines and oxaziridines for efficient, stable, and site-specific antibody bioconjugation. *Proc. Natl. Acad. Sci. 117*, 5733–5740.

(3) Hanwell, M. D., Curtis, D. E., Lonie, D. C., Vandermeersch, T., Zurek, E., and Hutchison, G. R. (2012) Avogadro: an advanced semantic chemical editor, visualization, and analysis platform. *J. Cheminformatics 4*, 17.

(4) Han, X., He, L., Xin, L., Shan, B., and Ma, B. (2011) PeaksPTM: Mass Spectrometry-Based Identification of Peptides with Unspecified Modifications. *J. Proteome Res. 10*, 2930–2936.

(5) Cho, H.-S., Mason, K., Ramyar, K. X., Stanley, A. M., Gabelli, S. B., Denney, D. W., and Leahy, D. J. (2003) Structure of the extracellular region of HER2 alone and in complex with the Herceptin Fab. *Nature 421*, 756–760.

(6) Cock, P. J. A., Antao, T., Chang, J. T., Chapman, B. A., Cox, C. J., Dalke, A., Friedberg, I., Hamelryck, T., Kauff, F., Wilczynski, B., and de Hoon, M. J. L. (2009) Biopython: freely available Python tools for computational molecular biology and bioinformatics. *Bioinforma. Oxf. Engl. 25*, 1422–1423.

(7) Hamelryck, T., and Manderick, B. (2003) PDB file parser and structure class implemented in Python. *Bioinformatics 19*, 2308–2310.

(8) MacLean, B., Tomazela, D. M., Shulman, N., Chambers, M., Finney, G. L., Frewen, B., Kern, R., Tabb, D. L., Liebler, D. C., and MacCoss, M. J. (2010) Skyline: an open source document editor for creating and analyzing targeted proteomics experiments. *Bioinforma. Oxf. Engl. 26*, 966–968.

(9) Geri, J. B., Oakley, J. V., Reyes-Robles, T., Wang, T., McCarver, S. J., White, C. H., Rodriguez-Rivera, F. P., Parker, D. L., Hett, E. C., Fadeyi, O. O., et al. (2020) Microenvironment mapping via Dexter energy transfer on immune cells. *Science 367*, 1091–1097.

(10) Cox, J., and Mann, M. (2008) MaxQuant enables high peptide identification rates, individualized p.p.b.-range mass accuracies and proteome-wide protein quantification. *Nat. Biotechnol. 26*, 1367–1372.

(11) Tyanova, S., Temu, T., and Cox, J. (2016) The MaxQuant computational platform for mass spectrometry-based shotgun proteomics. *Nat. Protoc. 11*, 2301–2319.

(12) Tyanova, S., Temu, T., Sinitcyn, P., Carlson, A., Hein, M. Y., Geiger, T., Mann, M., and Cox, J. (2016) The Perseus computational platform for comprehensive analysis of (prote)omics data. *Nat. Methods 13*, 731–740.

(13) Rudolph, J. D., and Cox, J. (2019) A Network Module for the Perseus Software for Computational Proteomics Facilitates Proteome Interaction Graph Analysis. *J. Proteome Res. 18*, 2052–2064.

(14) Waskom, M. L. (2021) seaborn: statistical data visualization. *J. Open Source Softw. 6*, 3021.
